# Supplementary material for: Cardiovascular risk factors and COVID-19 outcomes in hospitalised patients: a prospective cohort study
Source: BMJ Open. 2021 Feb 22;11(2):e045482. doi: 10.1136/bmjopen-2020-045482 (PMC7902321; doi:10.1136/bmjopen-2020-045482)
Supplement: Supplementary data [file bmjopen-2020-045482supp001.pdf]

Supplement 1: Baseline characteristics in subset with detailed clinical history

In three of the participating hospitals (Amsterdam UMC location AMC, Amsterdam UMC location VUMC, Flevoziekenhuis), we collected additional clinical information to validate the medication use as surrogate marker for cardiovascular risk factors and disease, depicted in the table below.

Supplementary table: Baseline characteristics of complete cohort, and subgroup where additional information about prior cardiovascular events was available.

	**Overall**	**Subset**
n	1604	566
Age (mean (SD))	65.67 (15.06)	61.37 (14.60)
Women	633 (39.5)	241 (42.6)
Chronic cardiac disease	467 (29.2)	125 (22.1)
Hypertension	734 (46.0)	252 (44.8)
Chronic pulmonary disease	288 (18.0)	77 (13.6)
Asthma	179 (11.2)	56 (9.9)
Chronic kidney disease	150 (9.4)	57 (10.1)
Diabetes	411 (25.7)	154 (27.2)
Malignant neoplasm	98 (6.2)	31 (5.5)
Chronic hematologic disorder	57 (3.6)	23 (4.1)
Smoking	78 (6.5)	28 (6.2)
Obesity	464 (30.8)	168 (32.8)
Combined use of beta-blockers and antiplatelet drugs	167 (10.4)	50 (8.8)
History of coronary artery disease		74 (13.1)
History of heart failure		21 (3.7)
History of stroke		42 (7.4)
History of peripheral artery disease		9 (1.6)
Antihypertensive-Rx		
0	883 (55.0)	327 (57.8)
1	377 (23.5)	126 (22.3)
2	282 (17.6)	81 (14.3)
≥ 3	62 (3.9)	32 (5.7)
Lipid-lowering-Rx		
0	1047 (65.3)	399 (70.5)
≥ 1	557 (34.7)	167 (29.5)
Glucose-lowering-Rx		
0	1250 (77.9)	435 (76.9)
1	170 (10.6)	60 (10.6)
≥ 2	184 (11.5)	71 (12.5)

RF, risk factor; SD, standard deviation; IQR, interquartile range, Rx medication.

Cox-regression for the effect of the number of antihypertensive, lipid-lowering and antidiabetic drug classes on mortality. Table depicts results from multivariate analysis

including age, sex, obesity, current smoker and the use of beta-blockers and antiplatelet medication. Rx, medication; HR, hazard ratio; CI, confidence interval.
