# Supplementary material for: Cardiovascular risk factors and COVID-19 outcomes in hospitalised patients: a prospective cohort study
Source: BMJ Open. 2021 Feb 22;11(2):e045482. doi: 10.1136/bmjopen-2020-045482 (PMC7902321; doi:10.1136/bmjopen-2020-045482)
Supplement: Supplementary data [file bmjopen-2020-045482supp002.pdf]

Supplement 2: Cox-regression models with additional correction for smoking, obesity and the use of both a beta-blocker and antiplatelet drug.

We performed an additional analysis for the association between the cardiovascular risk factors and mortality with adjustment for smoking, obesity and the use of both a beta-blocker and antiplatelet drug. Data for smoking status was missing for 22.7% of the patients, data for obesity was missing for 9.2% of the patients. Imputation for these covariates was performed using the Multivariate Imputation by Chained (mice) package version 3.8.0. The pooled results averaged over 25 iterations are depicted in the table below. A complete cases analysis showed similar results (data not shown).

Collard D, *et al. BMJ Open* 2021; 11:e045482. doi: 10.1136/bmjopen-2020-045482

BMJ Publishing Group Limited (BMJ) disclaims all liability and responsibility arising from any reliance placed on this supplemental material which has been supplied by the author(s)

Supplementary table: Effect of the cumulative risk factors, antihypertensive medication, lipid-lowering medication and antidiabetic medication on mortality after adjustment for covariates.

	Mortality			
**Covariate**	**HR**	**95%CI**		**P-value**
**1 RF**	1.01	0.73	1.39	0.956
**≥2 RF**	1.38	1.02	1.86	0.034
**Women**	0.94	0.74	1.19	0.612
**Age**	1.07	1.06	1.08	<0.001
**Beta-blockers and antiplatelet-Rx**	1.27	0.93	1.73	0.13
**Obesity**	1.26	0.97	1.65	0.086
**Current smoker**	0.83	0.46	1.5	0.533

	IC-admission			
**Covariate**	HR	95%CI		P-value
**1 RF**	1.06	0.77	1.47	0.723
**≥2 RF**	1.07	0.77	1.48	0.681
**Women**	0.45	0.34	0.59	<0.001
**Age**	0.99	0.98	1.00	0.023
**Beta-blockers and antiplatelet-Rx**	1.10	0.73	1.67	0.648
**Obesity**	1.31	1.01	1.71	0.042
**Current smoker**	0.66	0.37	1.19	0.167

	IC-mortality			
**Covariate**	HR	95%CI		P-value
**1 RF**	1.01	0.73	1.39	0.957
**≥2 RF**	1.38	1.02	1.86	0.035
**Women**	0.94	0.74	1.19	0.612
**Age**	1.07	1.06	1.08	<0.001
**Beta-blockers and antiplatelet-Rx**	1.27	0.93	1.73	0.131
**Obesity**	1.26	0.97	1.65	0.087
**Current smoker**	0.83	0.46	1.50	0.534

	Mortality			
**Covariate**	HR	95%CI		P-value
**1 BP-lowering-Rx**	1.04	0.78	1.38	0.79
**≥2 BP-lowering-Rx**	1.33	1.01	1.76	0.043
**Women**	0.9	0.72	1.14	0.391
**Age**	1.07	1.06	1.08	<0.001
**Beta-blockers and antiplatelet-Rx**	1.34	0.99	1.82	0.056
**Obesity**	1.27	0.97	1.66	0.078
**Current smoker**	0.87	0.5	1.51	0.615

	Mortality			
**Covariate**	HR	95%CI		P-value
**1 lipid-lowering-Rx**	1.14	0.89	1.45	0.292
**Women**	0.92	0.73	1.17	0.502
**Age**	1.07	1.06	1.08	<0.001
**Beta-blockers and antiplatelet-Rx**	1.36	1	1.86	0.051
**Obesity**	1.3	1	1.69	0.051
**Current smoker**	0.87	0.5	1.52	0.624

	Mortality			
**Covariate**	HR	95%CI		P-value
**1 antidiabetic-Rx**	1.25	0.9	1.74	0.182
**≥2 antidiabetic-Rx**	1.93	1.43	2.62	<0.001
**Women**	0.93	0.74	1.17	0.538
**Age**	1.07	1.06	1.08	<0.001
**Beta-blockers and antiplatelet-Rx**	1.34	0.99	1.8	0.057
**Obesity**	1.18	0.9	1.55	0.226
**Current smoker**	0.91	0.52	1.58	0.725
