# Supplementary material for: Cardiovascular risk factors and COVID-19 outcomes in hospitalised patients: a prospective cohort study
Source: BMJ Open. 2021 Feb 22;11(2):e045482. doi: 10.1136/bmjopen-2020-045482 (PMC7902321; doi:10.1136/bmjopen-2020-045482)
Supplement: Author's manuscript [file bmjopen-2020-045482.draft_revisions.pdf]

BMJ Open is committed to open peer review. As part of this commitment we make the peer review history of every article we publish publicly available.

When an article is published we post the peer reviewers' comments and the authors' responses online. We also post the versions of the paper that were used during peer review. These are the versions that the peer review comments apply to.

The versions of the paper that follow are the versions that were submitted during the peer review process. They are not the versions of record or the final published versions. They should not be cited or distributed as the published version of this manuscript.

BMJ Open is an open access journal and the full, final, typeset and author-corrected version of record of the manuscript is available on our site with no access controls, subscription charges or pay-per-view fees (http://bmjopen.bmj.com).

If you have any questions on BMJ Open's open peer review process please email info.bmjopen@bmj.com

Cardiovascular risk factors are independently associated with COVID-19 mortality: a prospective cohort study

Journal:	*BMJ Open*
Manuscript ID	bmjopen-2020-045482
Article Type:	Original research
Date Submitted by the Author:	06-Oct-2020
Complete List of Authors:	Collard, Didier; Amsterdam University Medical Centers, Vascular Medicine<br>Nurmohamed, Nick; Amsterdam University Medical Centers, Vascular Medicine<br>Kaiser, Yannick; Amsterdam University Medical Centers, Vascular Medicine<br>Reeskamp, Laurens; Amsterdam University Medical Centers, Vascular Medicine<br>Dormans, Tom; Zuyderland Medical Centre Sittard-Geleen, Department of Intensive Care<br>Moeniralam, Hazra; Sint Antonius Hospital, Internal Medicine<br>Simsek, Suat; Noordwest Ziekenhuisgroep, Department of Internal Medicine<br>Douma, Renee; Flevoziekenhuis, Internal Medicine<br>Eerens, Annet ; Treant Healthcare Group, Oncology<br>Reidinga, Auke; Martini Ziekenhuis, Intensive Care<br>Elbers, Paul; Amsterdam UMC - Locatie VUMC, Department of Intensive Care<br>Beudel, Martijn; Amsterdam University Medical Centers, Neurology<br>Vogt, Liffert; Amsterdam University Medical Centers, Internal Medicine<br>Stroes, Erik; Amsterdam University Medical Centers, Vascular Medicine<br>van den Born, Bert-Jan; Amsterdam University Medical Centers, Vascular Medicine
Keywords:	COVID-19, Hypertension < CARDIOLOGY, General diabetes < DIABETES & ENDOCRINOLOGY, INTENSIVE & CRITICAL CARE, Ischaemic heart disease < CARDIOLOGY, Vascular medicine < INTERNAL MEDICINE

SCHOLARONE™
Manuscripts

I, the Submitting Author has the right to grant and does grant on behalf of all authors of the Work (as defined in the below author licence), an exclusive licence and/or a non-exclusive licence for contributions from authors who are: i) UK Crown employees; ii) where BMJ has agreed a CC-BY licence shall apply, and/or iii) in accordance with the terms applicable for US Federal Government officers or employees acting as part of their official duties; on a worldwide, perpetual, irrevocable, royalty-free basis to BMJ Publishing Group Ltd ("BMJ") its licensees and where the relevant Journal is co-owned by BMJ to the co-owners of the Journal, to publish the Work in this journal and any other BMJ products and to exploit all rights, as set out in our licence.

The Submitting Author accepts and understands that any supply made under these terms is made by BMJ to the Submitting Author unless you are acting as an employee on behalf of your employer or a postgraduate student of an affiliated institution which is paying any applicable article publishing charge ("APC") for Open Access articles. Where the Submitting Author wishes to make the Work available on an Open Access basis (and intends to pay the relevant APC), the terms of reuse of such Open Access shall be governed by a Creative Commons licence – details of these licences and which Creative Commons licence will apply to this Work are set out in our licence referred to above.

Other than as permitted in any relevant BMJ Author's Self Archiving Policies, I confirm this Work has not been accepted for publication elsewhere, is not being considered for publication elsewhere and does not duplicate material already published. I confirm all authors consent to publication of this Work and authorise the granting of this licence.

For peer review only

Cardiovascular risk factors are independently associated with COVID-19 mortality: a prospective cohort study

Authors: Didier Collard MD[1*], Nick S. Nurmohamed MD[1,2*], Yannick Kaiser MD[1*], Laurens F. Reeskamp MD[1], Tom Dormans MD PhD[3], Hazra Moeniralam MD PhD[4], Suat Simsek MD PhD[5], Renée A. Douma MD PhD[6], Annet Eerens[7], Auke C. Reidinga MD[8], Paul Elbers MD PhD[9], Martijn Beudel MD PhD[10], Liffert Vogt MD PhD[11], Erik S.G. Stroes MD PhD[1], Bert-Jan H. van den Born MD PhD[1,12]

*These authors contributed equally to this work

Affiliations

[1] Amsterdam UMC, University of Amsterdam, Department of Vascular Medicine, Amsterdam Cardiovascular Sciences, Amsterdam, the Netherlands

[2] Amsterdam UMC, Vrije Universiteit Amsterdam, Department of Cardiology, Amsterdam Cardiovascular Sciences, Amsterdam, the Netherlands

[3] Zuyderland Medisch Centrum, Department of Intensive Care, Sittard-Geleen, the Netherlands

[4] St. Antonius Hospital, Department of Internal Medicine, Nieuwegein, the Netherlands

[5] Northwest Clinics, Department of Internal Medicine, Alkmaar, The Netherlands

[6] Flevohospital, Department of Internal Medicine, Almere, The Netherlands

[7] Treant Healthcare Group, Department of Oncology, Hoogeveen, The Netherlands

[8] Martini Hospital, Intensive Care Unit, Groningen, The Netherlands

[9] Amsterdam UMC, Vrije Universiteit Amsterdam, Department of Intensive Care, Amsterdam Amsterdam, the Netherlands

[10] Amsterdam UMC, University of Amsterdam, Department of Neurology, Amsterdam Neuroscience Institute, Amsterdam, the Netherlands

[11] Amsterdam UMC, University of Amsterdam, Department of Nephrology, Amsterdam Cardiovascular Sciences, Amsterdam, the Netherlands

[12] Amsterdam UMC, University of Amsterdam, Department of Public Health, Amsterdam, the Netherlands

Keywords: COVID-19, SARS-CoV-2, CVD, hypertension, diabetes, mortality, ICU-admission

Word count: 3562 (manuscript, including references), 291 (abstract)

Figures: tables: 1, figures: 1, supplementary figures: 1, supplementary tables: 2

Address for correspondence

Dr. Bert-Jan H. van den Born

Department of Vascular Medicine, Amsterdam University Medical Centers, University of Amsterdam

Meibergdreef 9, 1105 AZ Amsterdam, the Netherlands

Email: b.j.vandenborn@amsterdamumc.nl, tel: +3120-5666612

For peer review only - http://bmjopen.bmj.com/site/about/guidelines.xhtml

For peer review only

Abstract

Objectives: Recent reports suggest a high prevalence of hypertension and diabetes in COVID-19 patients, but the role of cardiovascular disease (CVD) risk factors in the clinical course of COVID-19 is unknown. We evaluated the time-to-event relationship between hypertension, dyslipidemia, diabetes, and COVID-19 outcomes.

Design: We analyzed data from the prospective Dutch COVID-PREDICT cohort, an ongoing prospective study of patients admitted for COVID-19 infection.

Setting: Patients from 8 participating hospitals, including two university hospitals from the COVID-PREDICT cohort were included.

Participants: Admitted, adult patients with a positive COVID-19 polymerase chain reaction (PCR) or high suspicion based on CT-imaging of the thorax. Patients were followed for major outcomes during hospitalization. CVD risk factors were established via home medication lists and divided in antihypertensives, lipid lowering therapy, and antidiabetics.

Primary and secondary outcomes measures: The primary outcome was mortality during the first 21 days following admission, secondary outcomes consisted of ICU-admission and ICU-mortality. Kaplan-Meier and Cox-regression analyses were used to determine the association with CVD risk factors.

Results: We included 1604 patients with a mean age of 66±15 of whom 60.5% were men. Antihypertensives, lipid lowering therapy, and antidiabetics were used by 45%, 34.7%, and 22.1% of patients. After adjustment for age and sex, the presence of ≥2 risk factors was associated with increased mortality risk (HR 1.52, 95%CI 1.15-2.02), but not with ICU-admission. Moreover, the use of ≥2 antidiabetics and ≥2 antihypertensives was associated with mortality independent of age and sex with HRs of respectively 2.09 (95%CI 1.55-2.80) and 1.46 (95%CI 1.11-1.91).

For peer review only - http://bmjopen.bmj.com/site/about/guidelines.xhtml

Conclusions: The accumulation of hypertension, dyslipidemia and diabetes leads to a stepwise increased risk for short-term mortality in hospitalized COVID-19 patients independent of age and sex. Further studies investigating how these risk factors disproportionately affect COVID-19 patients are warranted.

Strengths and limitations of this study

- While previous data reported a high prevalence of CVD risk factors in COVID-19 patients, this study investigated whether diabetes, dyslipidemia and hypertension predict adverse outcomes.
- This study is limited by the use of medication as surrogate for cardiovascular risk factors
- The causality of the investigated risk factors remains to be addressed in future studies.

For peer review only - http://bmjopen.bmj.com/site/about/guidelines.xhtml

Introduction

The global spread of coronavirus disease 2019 (COVID-19), first identified in Wuhan, China, in December 2019, has ignited an unprecedented ongoing global pandemic.(1) Although most infected individuals experience only mild symptoms that do not require hospitalization, the absolute number of patients requiring hospital admission is staggering. Risk stratification of these patients is crucial to optimize the use of hospital resources.(2) Several associations with adverse outcomes in COVID-19 patients have been identified, including factors that also predispose to cardiovascular disease (CVD), such as older age, male sex, hypertension, overweight and diabetes.(3,4) Furthermore, individuals with overt CVD appear to be affected more seriously by COVID-19 infection.(5)

The association between cardiovascular events and infectious diseases is well established. Examples include the increased prevalence of myocardial infarction during influenza pandemics,(6) and the higher number of cardiac complications in patients hospitalized for community-acquired pneumonia.(7) However, there are conflicting data whether the presence of shared CVD and COVID-19 risk factors merely reflect advanced age and history of ischemic heart disease in patients who develop severe infection, or are independently associated with adverse outcomes in the COVID-19 patient population.(4,8) For example, a higher than expected prevalence of diabetes, hypertension, obesity, and history of CVD was reported during the previous of outbreak Middle East respiratory syndrome coronavirus (MERS-CoV), which shares many similarities with COVID-19.(9)

In the present study, we hypothesized that three major risk independent CVD risk factors are associated with adverse outcomes in COVID-19 patients. To this end, we evaluated the time-to-event relationship between COVID-19 disease outcomes and a history of medication use for hypertension, dyslipidemia, and diabetes mellitus in a large prospective Dutch cohort of hospitalized COVID-19 patients.

For peer review only - http://bmjopen.bmj.com/site/about/guidelines.xhtml

Patients and Methods

Study design

COVID-PREDICT is a Dutch multicenter initiative to collect data of hospitalized patients with confirmed COVID-19. For this study, patients from 8 participating hospitals, including two university hospitals were included. All hospitalized patients >18 years with a positive COVID-19 polymerase chain reaction (PCR) or high suspicion based on CT-imaging of the thorax were included.(10) A waiver for the use hospital record data was obtained from the Medical Ethical Committees of the participating centers. Patients were given the opportunity to opt out.

The collected data was updated with daily reports on vital signs, laboratory results, complications, and clinical outcomes. In addition, the use of antihypertensive, lipid-lowering, and/or antidiabetic medication was determined from the home medication list. These were used as surrogates for hypertension, dyslipidemia, and diabetes. Antihypertensive medication was categorized as using either 0, 1, more than 1 of the following categories: non-dihydropyridine calcium channel blockers (CCBs), renin angiotensin system (RAS)-inhibitors (either angiotensin receptor antagonist or angiotensin receptor blockers) and diuretics (either loop diuretics, thiazide or thiazide like diuretics or potassium channel blockers). Lipid-lowering therapy was classified as the use of statin, ezetimibe, fibrates, or PCSK9-inhibitors. Antidiabetic medication was classified as using either 0, 1 or more than 1 of the following classes: metformin, sulfonylurea derivates, GLP-1 agonists, DDP4- inhibitors, SGLT-2 inhibitors, and insulin. Obesity was defined as a BMI >30 kg/m. Smoking was categorized into current or non/former smoker. The combined use of beta-blockers and platelet aggregation inhibitors was used as a surrogate for history of ischemic cardiac disease.

For peer review only - http://bmjopen.bmj.com/site/about/guidelines.xhtml

Outcomes were determined 3 and 6 weeks after admission, or earlier when the patient died or was discharged from the hospital.

Primary and secondary outcomes

The primary outcome consisted of overall mortality during the first 21 days following admission. Overall mortality was defined as either mortality during admission or discharge for palliative care, either at home or a palliative care facility. If the patient was discharged alive from the hospital and no further follow-up data was available, we considered the patient to be event free for the whole study period. Secondary outcomes consisted of ICU-admission and mortality in the subset of patients who had been admitted to the ICU.

Statistical analysis

For the analysis, we included all consecutive patients who were primarily admitted to one of the participating centers between February 27th and July 4th 2020. Patients with unknown medication use prior to hospitalization were excluded. Patients were categorized based on the presence of either 0, 1 or more than 1 medication based cardiovascular risk factor. Baseline characteristics were depicted as mean ± standard deviation for normally distributed data, nonnormally as median [interquartile range], or as number (percentage) for categorical variables, and were compared using the appropriate tests (ANOVA, Kruskal-Wallis, chi-squared). The relationship between outcomes and cumulative cardiovascular risk factors was determined using a Kaplan-Meier analysis. We then determined hazard ratios using Cox-regression after correction for age and sex, and with additional correcting for obesity, smoking and history of ischemic cardiac disease. For ICU-mortality, a landmark analysis was performed starting from ICU-admission. Next, with Cox-regression models using the same covariates we determined the association between mortality and the use of antihypertensive,

For peer review only

lipid-lowering, and antidiabetic drugs. All statistical analyses were conducted with R version 3.6.3 (R Foundation, Vienna, Austria) using the Survival version 3.1-11 and Tableone version 0.11.1 packages.

Results

Patient characteristics

Between February 27th 2020 and July 4th 2020, a total of 1614 patients with a confirmed COVID-19 infection were primarily admitted to one of the participating centers in the Netherlands. After exclusion of patients with an unknown medication list, we included 1604 patients in the present analysis. Their mean age was 66±15 years, 67.6% were Caucasian, and 60.5% were men. 6.5% of the admitted patients were current smokers. The majority of admitted patients (924, 57.6%) used some form of cardiovascular medication prior to admission. Antihypertensive medication was used by 721 (45.0%) patients, of whom 497 (68.9%) used RAS-inhibitors, 256 (35.5%) calcium-antagonists, and 374 (51.9%) diuretics. Lipid-lowering therapy was used by 557 (34.7%) patients, predominantly consisting of statins (540, 96.9%). In total, 354 (22.1%) patients used antidiabetic medication, of whom 282 (79.7%) used metformin and 137 patients (38.7%) insulin. 167 patients (10.4%) used the combination of a beta-blocker and a platelet aggregation inhibitor, reflecting a history of ischemic cardiac disease. In the subset of 566 patients for whom a more detailed medical history was available, we found a similar prevalence of cardiovascular disease, with 13.1% of the patients having a history of coronary artery disease, 3.7% of heart failure, 7.4% of stroke and 1.6% of peripheral arterial disease (see Supplement 3).

For peer review only - http://bmjopen.bmj.com/site/about/guidelines.xhtml

Cardiovascular risk factors as markers for mortality and ICU-admission

In the entire cohort, 308 (19.2%) of the patients died or were discharged for palliative care. In total 273 (17.0%) of the patients were admitted to the ICU, of whom 78 died. 1100 (68.6%) patients were discharged alive from the hospital, 50 (3.1%) were transferred to another hospital. The remaining patients were still admitted to the hospital at time of the data collection (126; 7.9%) or follow-up data was not available yet (20; 1.25%). The Kaplan-Meier analysis showed a significant association between cardiovascular risk factors and overall mortality ($p<0.0001$; Figure 1A) and a trend towards increased ICU-mortality ($p=0.055$; Supplement 1). We found no association between cardiovascular risk factors and ICU-admission ($p=0.85$; Figure 1B). In Cox-regression analysis, a 5-year age increase was associated with a HR of 1.37 (CI 1.31-1.45) for mortality, while there was no significant association with sex (HR 1.02, CI 0.81-1.28). The presence of two or more cardiovascular risk factors was significantly associated with overall mortality (HR 1.52, 95%CI 1.15-2.02), but not with ICU-admission or ICU-mortality (Table 2). After additional correction for smoking, obesity, and the combined use of beta-blockers and platelet aggregation inhibitors the presence of two or more risk factors remained associated with mortality (HR 1.38, 95%CI 1.02-1.86, Supplement 2).

Individual risk factors

In the Cox-regression models corrected for age and sex, we observed that the use of two or more different classes of antihypertensives and antidiabetics were associated with 21-day mortality, with HR of respectively of 1.46 (95%CI 1.11-1.91) and 2.09 (95%CI 1.55-2.80). Similarly, we found a HR of 1.25 (95%CI 0.99-1.56) for the use of lipid-lowering medication. Additional correction for smoking, obesity and the combined use of beta-blockers and antiplatelet medication attenuated the association between the use of BP-lowering and lipid-

For peer review only - http://bmjopen.bmj.com/site/about/guidelines.xhtml

lowering medication towards 1.33 (95%CI 1.01-1.76) and 1.14 (95%CI 0.89-1.45) for the use of ≥2 BP-lowering drugs or ≥1 lipid-lowering drug respectively. The association between the use of 2 or more glucose-lowering medications and mortality remained significant with an adjusted HR of 1.93 (95%CI 1.43-2.62; Supplementary table 2).

Discussion

In a large Dutch cohort of hospitalized COVID-19 patients, we observed that patients with more than one risk factor for CVD had a 52% higher 3-week mortality risk, independent of age and sex. In addition, our data show that the use of two or more antihypertensives or antidiabetics, or one lipid-lowering drug is associated with adverse outcomes in COVID-19 patients. Patients using two or more antidiabetic drugs had the highest mortality risk. This suggests that patients with a history of or at high risk for cardiovascular disease have an increased risk for adverse COVID-19 disease outcomes.

For peer review only

The prevalence of medication use for CVD risk factors was higher in COVID-19 patients than in previously described cohorts representative for the general Dutch population of similar age.(11) Wuhan-based COVID-19 cohorts were the first to describe a higher mortality in those with hypertension and diabetes.(12) However, the average age and reported prevalence of CVD risk factors was much lower in these cohorts than in the present study.(4) The increased case-fatality rate in Europe and the US compared with China may in part be attributable to demographic differences in the COVID-19 infected population.(13) Recent European and US-based cohorts indeed demonstrate a higher average age with a similar distribution of hypertension and diabetes compared to our study, and also show a higher mortality in COVID-19 patients with hypertension and diabetes.(14,15) In line with the results from a large US-based cohort, we observed that age was significantly associated with

For peer review only - http://bmjopen.bmj.com/site/about/guidelines.xhtml

mortality, while we found no significant difference in mortality between sexes.(16) This suggests that although men are more often hospitalized, there is no substantial difference in mortality after admission for severe COVID-19 infection. We add to these findings that the accumulation of CVD risk factors is associated with mortality, independent of age, sex, presence of coronary artery disease, smoking and obesity in hospitalized patients.

Analogous to COVID-19, CVD risk factors are also prevalent among patients hospitalized for community-acquired pneumonia.(17) However, we observed a stronger association between hypertension, diabetes, dyslipidemia and mortality in COVID-19 patients compared with previous studies on community-acquired pneumonia, despite a similar prevalence of CVD risk factors.(18) In addition, these effects remained significant after correction for covariates such as smoking, obesity, and the use of beta-blockers and antiplatelet drugs as surrogate for a history of ischemic cardiac disease. This might suggest that CVD risk factors in COVID-19 patients disproportionally affect the clinical course of COVID-19 patients compared to other infectious diseases. The present findings are comparable to the previous MERS-CoV outbreak, which also saw a preponderance of hypertension and diabetes in hospitalized patients.(9) Because both coronaviruses enter the cell through the angiotensin converting enzyme 2 (ACE2) receptor, a hypothesis has become that upregulation of ACE2 in patients with hypertension and/or diabetes facilitates transmission of the virus.(19–22) However, recent studies have shown no association between the use of RAS-medication and the disease course of COVID-19.(23,24) Furthermore, this theory does not explain the increased risk of mortality paired with other CVD risk factors in COVID-19 patients. An alternative explanation is that CVD risk factors predispose to myocardial injury in COVID-19 infected patients, contributing to a more severe clinical course.(25) Two recent studies revealed viral RNA in the myocardium of COVID-19 patients, suggesting that SARS-CoV-2 might infect

For peer review only - http://bmjopen.bmj.com/site/about/guidelines.xhtml
For peer review only

the heart directly. It can be speculated that those with (pre-clinical) atherosclerosis are prone to experience coronary ischemia from viral myocardial involvement.(26,27)

In our cohort of COVID-19 patients, the presence of diabetes had the strongest association with mortality and remained significant after correction for covariates such as smoking, obesity, and the use of beta-blockers and antiplatelet drugs as surrogate for coronary artery disease. We observed smaller effect sizes for the presence of hypertension and dyslipidemia. These findings might suggest that diabetes predisposes for adverse outcomes in COVID-19 patients not only through its association with CVD, but potentially via other pathophysiological pathways specific to diabetes. A recent Chinese cohort showed diabetes to be associated with higher ICU-admission and more in-hospital mortality, but not independently of hypertension and history of CVD.(8) Interestingly, in those with diabetes, hypertension was associated with in-hospital death, independently of history of CVD, further supporting the additive effect of CVD risk factors on COVID-19 mortality. Nevertheless, it is hard to disentangle the precise relation based on epidemiological data. In line with current guidelines on CVD risk management, cardiovascular medication from different classes were often prescribed together in our cohort, also in patients with diabetes.(28) This makes it difficult to assess their separate contribution to mortality. It remains, therefore, unknown whether diabetes alone is associated with a higher risk of adverse outcomes or whether it is merely a reflection of increased vascular ageing in combination with the other risk factors.(29) In contrast to our results, the recent cohort of Cummings et al, found that among cardiovascular risk factors, only chronic cardiac disease was a strong predictor for hospital mortality, while smaller associations were found for other risk factors, including diabetes.(16) In line, a recent Italian cohort of hospitalized COVID-19 patients showed an increased prevalence of hypertension and diabetes amongst non-survivors, but only diabetes was an

For peer review only - http://bmjopen.bmj.com/site/about/guidelines.xhtml

independent predictor after correction for other comorbidities.(30) In the present study, the cumulative presence of CVD risk factors did not show an association with increased risk for ICU-admission. This may have been influenced by selection prior to ICU admission, where the presence of co-morbidity was taken into account in the shared decision-making process, leading to a relative underrepresentation of patients with CV-risk factors.

The present analysis has several limitations. First, data collection was based on data collection forms of the WHO, which did not include detailed information on cardiovascular disease history. For this reason, we relied on medication use as a surrogate marker for established cardiovascular risk factors or disease, which has been used before in big cohort studies.(31) Nevertheless, some of these drugs might have been prescribed for different indications. Secondly, we only obtained follow-up during the first 21 days, however as depicted in the Kaplan-Meier analysis, almost all events occurred during the first 14 days, in line with earlier descriptions.(4) Finally, we cannot exclude that mortality in the current study is partially caused by other factors than COVID-19. However, as we used 21-day mortality as our primary outcome and only included patients admitted to the hospital with confirmed COVID-19 infection, it is very likely that the majority of deaths were directly attributable to COVID-19.

For peer review only

In conclusion, the accumulation of CVD risk factors leads to a stepwise increased risk for short-term mortality in hospitalized COVID-19 patients. Patients with diabetes had the highest risk, followed by similar risks for hypertension and dyslipidemia. Mechanistic studies investigating how CVD risk factors disproportionately affect COVID-19 patients compared to other infectious diseases are warranted.

For peer review only - http://bmjopen.bmj.com/site/about/guidelines.xhtml

Acknowledgements

We would like to thank the CovidPredict consortium (www.covidpredict.org) for their efforts in providing the patient data.

Author contributions

DC, NN, YK, LR, ES, BjvdB, LV, MB, PE conceptualized and designed the study. DC, NN, YK, LR performed the data analysis. DC, NN, YK, ES, BjvdB drafted the manuscript. All authors have made substantial contributions to the following: (1) the conception and design of the study, or acquisition of data, or analysis and interpretation of data, (2) revising the manuscript critically for important intellectual content, (3) final approval of the version to be submitted.

Funding

DC is supported by a ZonMW grant (project number: 10430022010002).

Competing interests

N.S.N. and L.F.R. are co-founders of Lipid Tools. E.S.G.S. reports personal fees from Amgen, personal fees from Sanofi-Regeneron, personal fees from Esperion, grants from Athera, outside the submitted work.

Data availability statement

The data that support the findings of this study are available from the corresponding author, upon reasonable request.

For peer review only - http://bmjopen.bmj.com/site/about/guidelines.xhtml

References

1. World Health Organization. Coronavirus disease 2019 (COVID-19), situation report 92.
2. Lipsitch M, Swerdlow DL, Finelli L. Defining the Epidemiology of Covid-19 - Studies Needed. N Engl J Med. 2020 Mar;382(13):1194–6.
3. Wilkins JT, Ning H, Berry J, Zhao L, Dyer AR, Lloyd-Jones DM. Lifetime risk and years lived free of total cardiovascular disease. JAMA. 2012;308(17):1795–801.
4. Zhou F, Yu T, Du R, Fan G, Liu Y, Liu Z, et al. Clinical course and risk factors for mortality of adult inpatients with COVID-19 in Wuhan, China: a retrospective cohort study. Lancet. 2020;
5. Zheng YY, Ma YT, Zhang JY, Xie X. COVID-19 and the cardiovascaular system. Nat Rev Cardiol. 2020 Mar;17:259–60.
6. Nguyen JL, Yang W, Ito K, Matte TD, Shaman J, Kinney PL. Seasonal influenza infections and cardiovascular disease mortality. JAMA Cardiol. 2016 Jun;1(3):274–81.
7. Corrales-Medina VF, Musher DM, Wells GA, Chirinos JA, Chen L, Fine MJ. Cardiac complications in patients with community-acquired pneumonia incidence, timing, risk factors, and association with short-term mortality. Circulation. 2012 Feb;125(6):773–81.
8. Shi Q, Zhang X, Jiang F, Zhang X, Hu N, Bimu C, et al. Clinical Characteristics and Risk Factors for Mortality of COVID-19 Patients With Diabetes in Wuhan, China: A Two-Center, Retrospective Study. Diabetes Care. 2020;43(7):1382–91.
9. Badawi A, Ryoo SG. Prevalence of comorbidities in the Middle East respiratory syndrome coronavirus (MERS-CoV): a systematic review and meta-analysis. Int J Infect Dis. 2016 Aug;49:129–33.
10. Bai HX, Hsieh B, Xiong Z, Halsey K, Choi JW, Tran TML, et al. Performance of

For peer review only

radiologists in differentiating COVID-19 from viral pneumonia on chest CT. Radiology. 2020;200823.

11. van Laer SD, Snijder MB, Agyemang C, Peters RJG, van den Born BJH. Ethnic differences in hypertension prevalence and contributing determinants – the HELIUS study. Eur J Prev Cardiol. 2018;25(18):1914–22.

12. Guan WJ, Ni ZY, Hu Y, Liang WH, Ou CQ, He JX, et al. Clinical Characteristics of Coronavirus Disease 2019 in China. N Engl J Med. 2020;1–13.

13. Onder G, Rezza G, Brusaferro S. Case-Fatality Rate and Characteristics of Patients Dying in Relation to COVID-19 in Italy. JAMA - J Am Med Assoc. 2020;2019:2019–20.

14. Richardson S, Hirsch JS, Narasimhan M, Crawford JM, McGinn T, Davidson KW, et al. Presenting Characteristics, Comorbidities, and Outcomes Among 5700 Patients Hospitalized With COVID-19 in the New York City Area. Jama. 2020;10022:1–8.

15. Goyal P, Choi JJ, Pinheiro LC, Schenck EJ, Chen R, Jabri A, et al. Clinical Characteristics of Covid-19 in New York City. N Engl J Med. 2020 Apr 17;

16. Cummings MJ, Baldwin MR, Abrams D, Jacobson SD, Meyer BJ, Balough EM, et al. Epidemiology, clinical course, and outcomes of critically ill adults with COVID-19 in New York City: a prospective cohort study. Lancet [Internet]. 2020;S0140-6736(20)31189-2. Available from: http://dx.doi.org/10.1016/S0140-6736(20)31189-2

17. Corrales-Medina VF, Musher DM, Shachkina S, Chirinos JA. Acute pneumonia and the cardiovascular system. Lancet. 2013;381(9865):496–505.

18. Violi F, Cangemi R, Falcone M, Taliani G, Pieralli F, Vannucchi V, et al. Cardiovascular complications and short-term mortality risk in community-acquired pneumonia. Clin Infect Dis. 2017;64(11):1486–93.

19. Fang L, Karakiulakis G, Roth M. Are patients with hypertension and diabetes mellitus

For peer review only

at increased risk for COVID-19 infection? Lancet Respir Med. 2020 Apr;8(4):e21.

20. Vaduganathan M, Vardeny O, Michel T, McMurray JJ V, Pfeffer MA, Solomon SD. Renin-Angiotensin-Aldosterone System Inhibitors in Patients with Covid-19. N Engl J Med. 2020 Mar;

21. Kuster GM, Pfister O, Burkard T, Zhou Q, Twerenbold R, Haaf P, et al. SARS-CoV2: should inhibitors of the renin–angiotensin system be withdrawn in patients with COVID-19? Eur Heart J. 2020 Mar;

22. Danser AHHJ, Epstein M, Batlle D. Renin-Angiotensin System Blockers and the COVID-19 Pandemic: At Present There Is No Evidence to Abandon Renin-Angiotensin System Blockers. Hypertension. 2020 Mar;

23. Mackey K, King VJ, Gurley S, Kiefer M, Liederbauer E, Vela K, et al. Risks and Impact of Angiotensin-Converting Enzyme Inhibitors or Angiotensin-Receptor Blockers on SARS-CoV-2 Infection in Adults. Ann Intern Med [Internet]. 2020 May 15; Available from: https://doi.org/10.7326/M20-1515

24. Mancia G, Rea F, Ludergnani M, Apolone G, Corrao G. Renin–angiotensin–aldosterone system blockers and the risk of COVID-19. N Engl J Med. 2020;382(25):2431–40.

25. Shi S, Qin M, Shen B, Cai Y, Liu T, Yang F, et al. Association of Cardiac Injury with Mortality in Hospitalized Patients with COVID-19 in Wuhan, China. JAMA Cardiol. 2020;

26. Wichmann D, Sperhake J-P, Lütgehetmann M, Steurer S, Edler C, Heinemann A, et al. Autopsy Findings and Venous Thromboembolism in Patients With COVID-19. Ann Intern Med [Internet]. 2020 May 6; Available from: https://doi.org/10.7326/M20-2003

27. Schaller T, Hirschbühl K, Burkhardt K, Braun G, Trepel M, Märkl B, et al. Postmortem Examination of Patients with COVID-19. JAMA - J Am Med Assoc.

For peer review only - http://bmjopen.bmj.com/site/about/guidelines.xhtml

2020;323(24):2518–20.

28. The Task Force for the management of arterial hypertension of the European Society of Cardiology (ESC) and the European Society of Hypertension (ESH). 2018 ESC/ESH Guidelines for the management of arterial hypertension. Eur Heart J. 2018 Aug 25;39(33):3021–104.

29. Ungvari Z, Tarantini S, Donato AJ, Galvan V, Csiszar A. Mechanisms of vascular aging. Circ Res. 2018;123(7):849–67.

30. Iaccarino G, Grassi G, Borghi C, Ferri C, Salvetti M, Volpe M. Age and Multimorbidity Predict Death Among COVID-19 Patients: Results of the SARS-RAS Study of the Italian Society of Hypertension. Hypertens (Dallas, Tex 1979). 2020;76(2):366–72.

31. Attaei MW, Khatib R, McKee M, Lear S, Dagenais G, Igumbor EU, et al. Availability and affordability of blood pressure-lowering medicines and the effect on blood pressure control in high-income, middle-income, and low-income countries: an analysis of the PURE study data. Lancet Public Heal. 2017;2(9):e411–9.

For peer review only

Figure legends

Figure 1. Survival and time-to event analysis of cumulative cardiovascular risk factors on mortality and ICU-admission

Kaplan-Meier analysis of hypertension, dyslipidemia and diabetes stratified into 0, 1 or more risk factors versus adverse clinical outcomes. Left panel (A) depicts mortality, right panel (B) ICU-admission. Log-rank test was used to test for differences between curves. RF, risk factor; ICU, intensive care unit.

For peer review only

For peer review only - http://bmjopen.bmj.com/site/about/guidelines.xhtml

Table 1. Baseline characteristics

	Overall	0 RF	1 RF	≥2 RF	p
n	1604	680	400	524	
Age (mean (SD))	65.67 (15.06)	58.59 (15.71)	69.93 (13.01)	71.62 (11.45)	<0.001
Women	633 (39.5)	298 (43.8)	170 (42.5)	165 (31.5)	<0.001
Chronic cardiac disease	467 (29.2)	71 (10.5)	117 (29.3)	279 (53.4)	<0.001
Hypertension	734 (46.0)	80 (11.8)	242 (60.8)	412 (78.8)	<0.001
Chronic pulmonary disease	288 (18.0)	92 (13.6)	82 (20.6)	114 (21.9)	<0.001
Asthma	179 (11.2)	77 (11.4)	41 (10.3)	61 (11.7)	0.793
Chronic kidney disease	150 (9.4)	17 (2.5)	48 (12.0)	85 (16.3)	<0.001
Diabetes	411 (25.7)	19 (2.8)	66 (16.5)	326 (62.3)	<0.001
Malignant neoplasm	98 (6.2)	36 (5.3)	25 (6.3)	37 (7.1)	0.455
Chronic hematologic disorder	57 (3.6)	32 (4.7)	11 (2.8)	14 (2.7)	0.1
Smoking	78 (6.5)	30 (6.0)	16 (5.4)	32 (7.8)	0.383
Obesity	464 (30.8)	164 (26.1)	112 (29.4)	188 (37.8)	<0.001
Combined use of beta-blockers and antiplatelet drugs	167 (10.4)	7 (1.0)	35 (8.8)	125 (23.9)	<0.001
Antihypertensive-Rx					<0.001
0	883 (55.0)	680 (100.0)	145 (36.2)	58 (11.1)	
1	377 (23.5)	0 (0.0)	141 (35.2)	236 (45.0)	
2	282 (17.6)	0 (0.0)	95 (23.8)	187 (35.7)	
≥ 3	62 (3.9)	0 (0.0)	19 (4.8)	43 (8.2)	
Lipid-lowering-Rx					<0.001
0	1047 (65.3)	0 (0.0)	296 (74.0)	89 (13.5)	
≥ 1	557 (34.7)	0 (0.0)	104 (26.0)	453 (86.5)	
Glucose-lowering-Rx					<0.001
0	1250 (77.9)	680 (100.0)	359 (89.8)	211 (40.3)	
1	170 (10.6)	0 (0.0)	23 (5.8)	147 (28.1)	
≥ 2	184 (11.5)	0 (0.0)	18 (4.5)	166 (31.7)	

RF, risk factor; SD, standard deviation; IQR, interquartile range; Rx, medication. P-values indicate comparison between subgroups based on the presence of cumulative risk factors.

For peer review only - http://bmjopen.bmj.com/site/about/guidelines.xhtml

Table 2. Effect of cumulative cardiovascular risk factors on primary and secondary outcomes

	Cumulative risk factors	HR	95%CI		p
Mortality	0 RF	1 (ref)			
	1 RF	1.04	0.76	1.43	0.786
	≥2 RF	1.52	1.15	2.02	0.004
ICU admission	0 RF	1 (ref)			
	1 RF	1.11	0.80	1.53	0.534
	≥2 RF	1.15	0.85	1.56	0.355
ICU-mortality	0 RF	1 (ref)			
	1 RF	0.86	0.46	1.60	0.625
	≥2 RF	1.52	0.90	2.55	0.115

Cox-regression for the effect of cumulative cardiovascular risk factors on mortality, ICU-admission and ICU-mortality, corrected for sex and age. HR, hazard ratio; CI, confidence interval; ICU, intensive care unit.

For peer review only

For peer review only - http://bmjopen.bmj.com/site/about/guidelines.xhtml

Table 3. Effect of antihypertensive, lipid-lowering, and antidiabetic medications on mortality

**Mortality**	**HR**	**95%CI**		**p**
0 antihypertensive-Rx	1 (ref)			
1 antihypertensive-Rx	1.08	0.81	1.43	0.597
≥2 antihypertensive-Rx	1.46	1.11	1.91	0.006
0 lipid-lowering-Rx	1 (ref)			
≥1 lipid-lowering-Rx	1.25	0.99	1.56	0.058
0 antidiabetic-Rx	1 (ref)			
1 antidiabetic-Rx	1.34	0.97	1.85	0.077
≥2 antidiabetic-Rx	2.09	1.55	2.80	<0.001

Cox-regression for the effect of the number of antihypertensive, lipid-lowering and antidiabetic drug classes on mortality, with correction for age and sex. Rx, medication; HR, hazard ratio; CI, confidence interval.

For peer review only

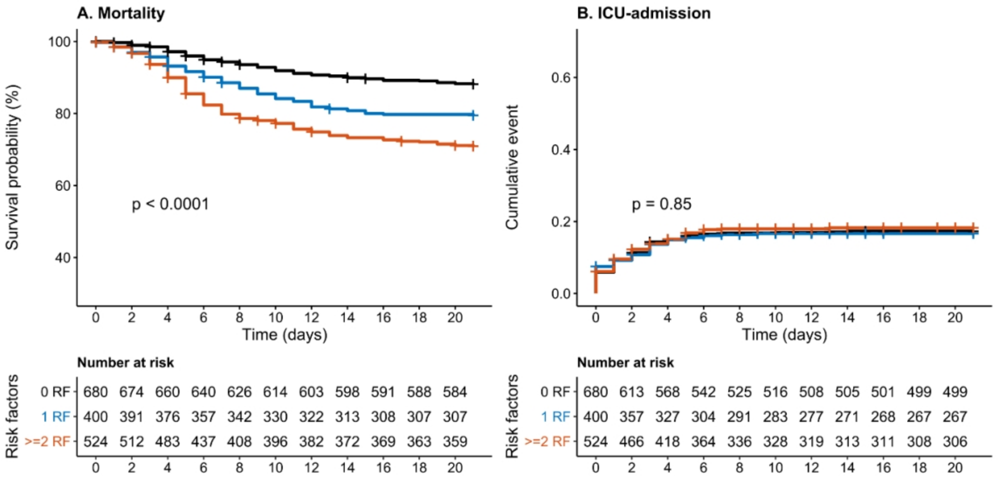

Figure 1. Kaplan-Meier analysis of hypertension, dyslipidemia and diabetes stratified into 0, 1 or more risk factors versus adverse clinical outcomes. Left panel (A) depicts mortality, right panel (B) ICU-admission. Log-rank test was used to test for differences between curves. RF, risk factor; ICU, intensive care unit.

117x58mm (300 x 300 DPI)

Supplement 1. Survival analysis for ICU-mortality

Figure S1. Survival analysis of cumulative cardiovascular risk factors for ICU-mortality

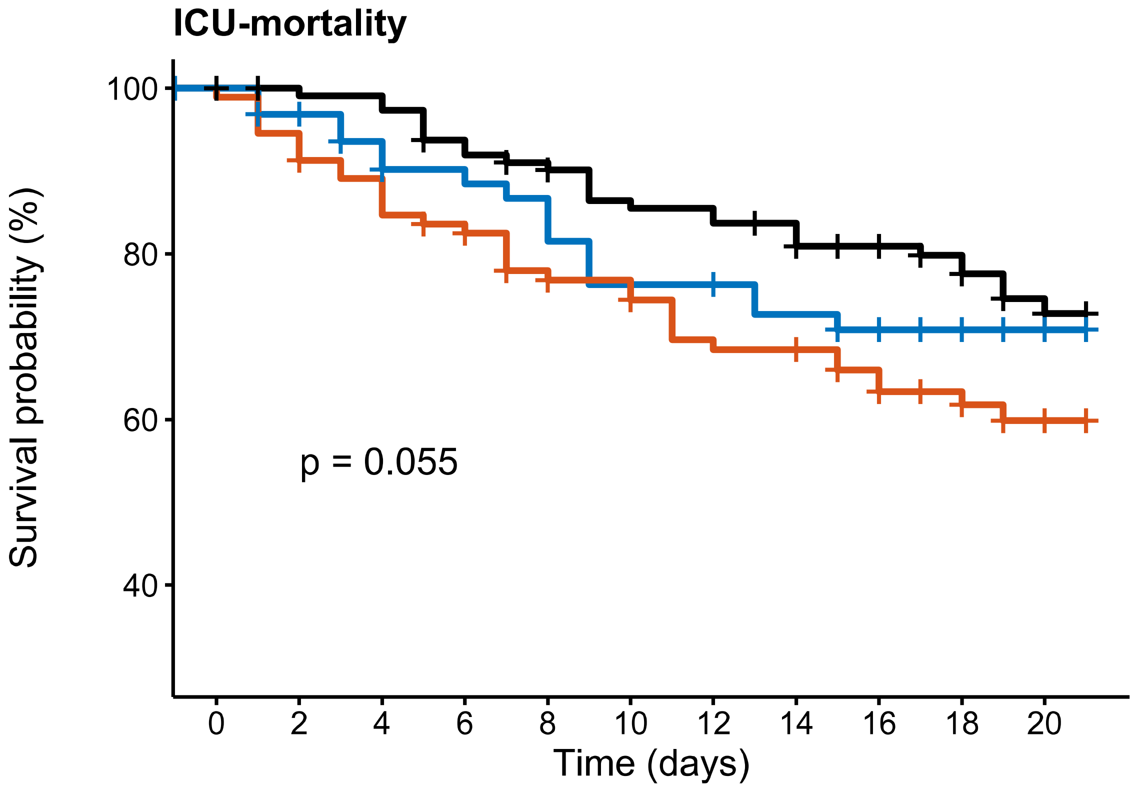

Number at risk

Risk factors	0	2	4	6	8	10	12	14	16	18	20
0 RF	115	112	111	104	100	94	93	90	81	71	41
1 RF	64	60	55	52	50	44	44	40	38	34	22
>=2 RF	92	87	81	75	68	65	58	57	50	40	25

Kaplan-Meier analysis of hypertension, dyslipidemia and diabetes stratified into 0, 1 or more risk factors versus adverse clinical outcomes for mortality in patients admitted to the ICU. Log-rank test was used to test for differences between curves. RF, risk factor; ICU, intensive care unit.

Supplement 2: Cox-regression models with additional correction for smoking, obesity and the use of both a beta-blocker and antiplatelet drug.

We performed an additional analysis for the association between the cardiovascular risk factors and mortality with correction for smoking, obesity and the use of both a beta-blocker and antiplatelet drug. Data for smoking status was missing for 22.7% of the patients, data for obesity was missing for 9.2% of the patients. Imputation for these covariates was performed using the Multivariate Imputation by Chained (mice) package version 3.8.0. The pooled results averaged over 25 iterations are depicted in Supplementary Table 2. A complete cases analysis showed similar results (data not shown).

For peer review only

For peer review only - http://bmjopen.bmj.com/site/about/guidelines.xhtml

Supplementary table S1: Effect of the cumulative risk factors, antihypertensive medication, lipid-lowering medication and antidiabetic medication on mortality after correction for covariates.

	Mortality			
**Covariate**	**HR**	**95%CI**		**P-value**
**1 RF**	1.01	0.73	1.39	0.956
**≥2 RF**	1.38	1.02	1.86	0.034
**Women**	0.94	0.74	1.19	0.612
**Age**	1.07	1.06	1.08	<0.001
**Beta-blockers and antiplatelet-Rx**	1.27	0.93	1.73	0.130
**Obesity**	1.26	0.97	1.65	0.086
**Current smoker**	0.83	0.46	1.50	0.533

	Mortality					Mortality					Mortality			
**Covariate**	HR	95%CI		P-value	**Covariate**	HR	95%CI		P-value	**Covariate**	HR	95%CI		P-value
**1 BP-lowering-Rx**	1.04	0.78	1.38	0.790	**1 lipid-lowering-Rx**	1.14	0.89	1.45	0.292	**1 antidiabetic-Rx**	1.25	0.90	1.74	0.182
**≥2 BP-lowering-Rx**	1.33	1.01	1.76	0.043						**≥2 antidiabetic-Rx**	1.93	1.43	2.62	<0.001
**Women**	0.90	0.72	1.14	0.391	**Women**	0.92	0.73	1.17	0.502	**Women**	0.93	0.74	1.17	0.538
**Age**	1.07	1.06	1.08	<0.001	**Age**	1.07	1.06	1.08	<0.001	**Age**	1.07	1.06	1.08	<0.001
**Beta-blockers and antiplatelet-Rx**	1.34	0.99	1.82	0.056	**Beta-blockers and antiplatelet-Rx**	1.36	1.00	1.86	0.051	**Beta-blockers and antiplatelet-Rx**	1.34	0.99	1.80	0.057
**Obesity**	1.27	0.97	1.66	0.078	**Obesity**	1.30	1.00	1.69	0.051	**Obesity**	1.18	0.90	1.55	0.226
**Current smoker**	0.87	0.50	1.51	0.615	**Current smoker**	0.87	0.50	1.52	0.624	**Current smoker**	0.91	0.52	1.58	0.725

For peer review only

For peer review only - http://bmjopen.bmj.com/site/about/guidelines.xhtml

Supplement 3: Baseline characteristics in subset with detailed clinical history

In three of the participating hospitals (Amsterdam UMC location AMC, Amsterdam UMC location VUMC, Flevoziekenhuis), we collected additional clinical information to validate the medication use as surrogate marker for cardiovascular risk factors and disease.

For peer review only

Supplementary table S2: Baseline characteristics of complete cohort, and subgroup where additional information about prior cardiovascular events was available.

		Overall	Subset
n		1604	566
Age (mean (SD))		65.67 (15.06)	61.37 (14.60)
Women		633 (39.5)	241 (42.6)
Chronic cardiac disease		467 (29.2)	125 (22.1)
Hypertension		734 (46.0)	252 (44.8)
Chronic pulmonary disease		288 (18.0)	77 (13.6)
Asthma		179 (11.2)	56 (9.9)
Chronic kidney disease		150 (9.4)	57 (10.1)
Diabetes		411 (25.7)	154 (27.2)
Malignant neoplasm		98 (6.2)	31 (5.5)
Chronic hematologic disorder		57 (3.6)	23 (4.1)
Smoking		78 (6.5)	28 (6.2)
Obesity		464 (30.8)	168 (32.8)
Combined use of beta-blockers and antiplatelet drugs		167 (10.4)	50 (8.8)
History of coronary artery disease			74 (13.1)
History of heart failure			21 (3.7)
History of stroke			42 (7.4)
History of peripheral artery disease			9 (1.6)
Antihypertensive-Rx			
	0	883 (55.0)	327 (57.8)
	1	377 (23.5)	126 (22.3)
	2	282 (17.6)	81 (14.3)
	≥ 3	62 (3.9)	32 (5.7)
Lipid-lowering-Rx			
	0	1047 (65.3)	399 (70.5)
	≥ 1	557 (34.7)	167 (29.5)
Glucose-lowering-Rx			
	0	1250 (77.9)	435 (76.9)
	1	170 (10.6)	60 (10.6)
	≥ 2	184 (11.5)	71 (12.5)

RF, risk factor; SD, standard deviation; IQR, interquartile range, Rx medication.

Cox-regression for the effect of the number of antihypertensive, lipid-lowering and antidiabetic drug classes on mortality. Table depicts results from multivariate analysis

For peer review only

including age, sex, obesity, current smoker and the use of beta-blockers and antiplatelet medication. Rx, medication; HR, hazard ratio; CI, confidence interval.

For peer review only

STROBE 2007 (v4) Statement—Checklist of items that should be included in reports of *cohort studies*

**Section/Topic**	**Item #**	**Recommendation**	**Reported on page #**
**Title and abstract**	1	(*a*) Indicate the study's design with a commonly used term in the title or the abstract	1
		(*b*) Provide in the abstract an informative and balanced summary of what was done and what was found	2
**Introduction**			
Background/rationale	2	Explain the scientific background and rationale for the investigation being reported	4
Objectives	3	State specific objectives, including any prespecified hypotheses	4
**Methods**			
Study design	4	Present key elements of study design early in the paper	5
Setting	5	Describe the setting, locations, and relevant dates, including periods of recruitment, exposure, follow-up, and data collection	5
Participants	6	(*a*) Give the eligibility criteria, and the sources and methods of selection of participants. Describe methods of follow-up	5
		(*b*) For matched studies, give matching criteria and number of exposed and unexposed	
Variables	7	Clearly define all outcomes, exposures, predictors, potential confounders, and effect modifiers. Give diagnostic criteria, if applicable	6
Data sources/ measurement	8*	For each variable of interest, give sources of data and details of methods of assessment (measurement). Describe comparability of assessment methods if there is more than one group	5
Bias	9	Describe any efforts to address potential sources of bias	6
Study size	10	Explain how the study size was arrived at	6
Quantitative variables	11	Explain how quantitative variables were handled in the analyses. If applicable, describe which groupings were chosen and why	6
Statistical methods	12	(*a*) Describe all statistical methods, including those used to control for confounding	6
		(*b*) Describe any methods used to examine subgroups and interactions	6
		(*c*) Explain how missing data were addressed	6; 23
		(*d*) If applicable, explain how loss to follow-up was addressed	6
		(*e*) Describe any sensitivity analyses	6
**Results**			

For peer review only

Participants	13*	(a) Report numbers of individuals at each stage of study—eg numbers potentially eligible, examined for eligibility, confirmed eligible, included in the study, completing follow-up, and analysed	7
		(b) Give reasons for non-participation at each stage	7
		(c) Consider use of a flow diagram	7
Descriptive data	14*	(a) Give characteristics of study participants (eg demographic, clinical, social) and information on exposures and potential confounders	7, 19, 27
		(b) Indicate number of participants with missing data for each variable of interest	23
		(c) Summarise follow-up time (eg, average and total amount)	6, 18, 22
Outcome data	15*	Report numbers of outcome events or summary measures over time	8
Main results	16	(*a*) Give unadjusted estimates and, if applicable, confounder-adjusted estimates and their precision (eg, 95% confidence interval). Make clear which confounders were adjusted for and why they were included	8,20, 21, 24
		(*b*) Report category boundaries when continuous variables were categorized	
		(*c*) If relevant, consider translating estimates of relative risk into absolute risk for a meaningful time period	
Other analyses	17	Report other analyses done—eg analyses of subgroups and interactions, and sensitivity analyses	8, 22
**Discussion**			
Key results	18	Summarise key results with reference to study objectives	9
**Limitations**			
Interpretation	20	Give a cautious overall interpretation of results considering objectives, limitations, multiplicity of analyses, results from similar studies, and other relevant evidence	9,10,11,12
Generalisability	21	Discuss the generalisability (external validity) of the study results	9,10,11,12
**Other information**			
Funding	22	Give the source of funding and the role of the funders for the present study and, if applicable, for the original study on which the present article is based	13

*Give information separately for cases and controls in case-control studies and, if applicable, for exposed and unexposed groups in cohort and cross-sectional studies.

Note: An Explanation and Elaboration article discusses each checklist item and gives methodological background and published examples of transparent reporting. The STROBE checklist is best used in conjunction with this article (freely available on the Web sites of PLoS Medicine at http://www.plosmedicine.org/, Annals of Internal Medicine at http://www.annals.org/, and Epidemiology at http://www.epidem.com/). Information on the STROBE Initiative is available at www.strobe-statement.org.

For peer review only

BMJ Open

Cardiovascular risk factors and COVID-19 outcomes in hospitalized patients: a prospective cohort study

Journal:	*BMJ Open*
Manuscript ID	bmjopen-2020-045482.R1
Article Type:	Original research
Date Submitted by the Author:	24-Nov-2020
Complete List of Authors:	Collard, Didier; Amsterdam UMC Location AMC, Vascular Medicine<br>Nurmohamed, Nick; Amsterdam UMC Location AMC, Vascular Medicine; Amsterdam UMC Locatie VUmc, Cardiology<br>Kaiser, Yannick; Amsterdam UMC Location AMC, Vascular Medicine<br>Reeskamp, Laurens; Amsterdam University Medical Centers, Vascular Medicine<br>Dormans, Tom; Zuyderland Medical Centre Sittard-Geleen, Department of Intensive Care<br>Moeniralam, Hazra; Sint Antonius Hospital, Internal Medicine<br>Simsek, Suat; Noordwest Ziekenhuisgroep, Department of Internal Medicine<br>Douma, Renee; Flevoziekenhuis, Internal Medicine<br>Eerens, Annet ; Treant Healthcare Group, Oncology<br>Reidinga, Auke; Martini Ziekenhuis, Intensive Care<br>Elbers, Paul; Amsterdam UMC - Locatie VUMC, Department of Intensive Care<br>Beudel, Martijn; Amsterdam UMC Location AMC, Neurology<br>Vogt, Liffert; Amsterdam UMC Location AMC, Internal Medicine<br>Stroes, Erik; Amsterdam UMC Location AMC, Vascular Medicine<br>van den Born, Bert-Jan; Amsterdam UMC Location AMC, Vascular Medicine
**Primary Subject Heading**:	Infectious diseases
Secondary Subject Heading:	Intensive care, Cardiovascular medicine, Epidemiology
Keywords:	COVID-19, Hypertension < CARDIOLOGY, General diabetes < DIABETES & ENDOCRINOLOGY, INTENSIVE & CRITICAL CARE, Ischaemic heart disease < CARDIOLOGY, Vascular medicine < INTERNAL MEDICINE

SCHOLARONE™
Manuscripts

I, the Submitting Author has the right to grant and does grant on behalf of all authors of the Work (as defined in the below author licence), an exclusive licence and/or a non-exclusive licence for contributions from authors who are: i) UK Crown employees; ii) where BMJ has agreed a CC-BY licence shall apply, and/or iii) in accordance with the terms applicable for US Federal Government officers or employees acting as part of their official duties; on a worldwide, perpetual, irrevocable, royalty-free basis to BMJ Publishing Group Ltd ("BMJ") its licensees and where the relevant Journal is co-owned by BMJ to the co-owners of the Journal, to publish the Work in this journal and any other BMJ products and to exploit all rights, as set out in our licence.

The Submitting Author accepts and understands that any supply made under these terms is made by BMJ to the Submitting Author unless you are acting as an employee on behalf of your employer or a postgraduate student of an affiliated institution which is paying any applicable article publishing charge ("APC") for Open Access articles. Where the Submitting Author wishes to make the Work available on an Open Access basis (and intends to pay the relevant APC), the terms of reuse of such Open Access shall be governed by a Creative Commons licence – details of these licences and which Creative Commons licence will apply to this Work are set out in our licence referred to above.

Other than as permitted in any relevant BMJ Author's Self Archiving Policies, I confirm this Work has not been accepted for publication elsewhere, is not being considered for publication elsewhere and does not duplicate material already published. I confirm all authors consent to publication of this Work and authorise the granting of this licence.

For peer review only

Cardiovascular risk factors and COVID-19 outcomes in hospitalized patients: a prospective cohort study

Authors: Didier Collard MD[1*], Nick S. Nurmohamed MD[1,2*], Yannick Kaiser MD[1*], Laurens F. Reeskamp MD[1], Tom Dormans MD PhD[3], Hazra Moeniralam MD PhD[4], Suat Simsek MD PhD[5], Renée A. Douma MD PhD[6], Annet Eerens[7], Auke C. Reidinga MD[8], Paul Elbers MD PhD[9], Martijn Beudel MD PhD[10], Liffert Vogt MD PhD[11], Erik S.G. Stroes MD PhD[1], Bert-Jan H. van den Born MD PhD[1,12]

*These authors contributed equally to this work

Affiliations

[1] Amsterdam UMC, University of Amsterdam, Department of Vascular Medicine, Amsterdam Cardiovascular Sciences, Amsterdam, the Netherlands

[2] Amsterdam UMC, Vrije Universiteit Amsterdam, Department of Cardiology, Amsterdam Cardiovascular Sciences, Amsterdam, the Netherlands

[3] Zuyderland Medisch Centrum, Department of Intensive Care, Sittard-Geleen, the Netherlands

[4] St. Antonius Hospital, Department of Internal Medicine, Nieuwegein, the Netherlands

[5] Northwest Clinics, Department of Internal Medicine, Alkmaar, The Netherlands

[6] Flevohospital, Department of Internal Medicine, Almere, The Netherlands

[7] Treant Healthcare Group, Department of Oncology, Hoogeveen, The Netherlands

[8] Martini Hospital, Intensive Care Unit, Groningen, The Netherlands

[9] Amsterdam UMC, Vrije Universiteit Amsterdam, Department of Intensive Care, Amsterdam Amsterdam, the Netherlands

[10] Amsterdam UMC, University of Amsterdam, Department of Neurology, Amsterdam Neuroscience Institute, Amsterdam, the Netherlands

[11] Amsterdam UMC, University of Amsterdam, Department of Nephrology, Amsterdam Cardiovascular Sciences, Amsterdam, the Netherlands

[12] Amsterdam UMC, University of Amsterdam, Department of Public Health, Amsterdam, the Netherlands

Keywords: COVID-19, SARS-CoV-2, CVD, hypertension, diabetes, mortality, ICU-admission

Word count: 3724 (manuscript, including references), 300 (abstract)

Figures: tables: 3, figures: 1, supplements: 2

Address for correspondence

Dr. Bert-Jan H. van den Born

Department of Vascular Medicine, Amsterdam University Medical Centers, University of Amsterdam

Meibergdreef 9, 1105 AZ Amsterdam, the Netherlands

Email: b.j.vandenborn@amsterdamumc.nl, tel: +3120-5666612

For peer review only - http://bmjopen.bmj.com/site/about/guidelines.xhtml

Abstract

Objectives: Recent reports suggest a high prevalence of hypertension and diabetes in COVID-19 patients, but the role of cardiovascular disease (CVD) risk factors in the clinical course of COVID-19 is unknown. We evaluated the time-to-event relationship between hypertension, dyslipidemia, diabetes, and COVID-19 outcomes.

Design: We analyzed data from the prospective Dutch COVID-PREDICT cohort, an ongoing prospective study of patients admitted for COVID-19 infection.

Setting: Patients from 8 participating hospitals, including two university hospitals from the COVID-PREDICT cohort were included.

Participants: Admitted, adult patients with a positive COVID-19 polymerase chain reaction (PCR) or high suspicion based on CT-imaging of the thorax. Patients were followed for major outcomes during hospitalization. CVD risk factors were established via home medication lists and divided in antihypertensives, lipid lowering therapy, and antidiabetics.

Primary and secondary outcomes measures: The primary outcome was mortality during the first 21 days following admission, secondary outcomes consisted of ICU-admission and ICU-mortality. Kaplan-Meier and Cox-regression analyses were used to determine the association with CVD risk factors.

Results: We included 1604 patients with a mean age of 66±15 of whom 60.5% were men. Antihypertensives, lipid lowering therapy, and antidiabetics were used by 45%, 34.7%, and 22.1% of patients. After 21-days of follow-up; 19.2% of the patients had died or were discharged for palliative care. Cox-regression analysis after adjustment for age and sex showed that the presence of ≥2 risk factors was associated with increased mortality risk (HR 1.52, 95%CI 1.15-2.02), but not with ICU-admission. Moreover, the use of ≥2 antidiabetics and ≥2 antihypertensives was associated with mortality independent of age and sex with HRs of respectively 2.09 (95%CI 1.55-2.80) and 1.46 (95%CI 1.11-1.91).

For peer review only

Conclusions: The accumulation of hypertension, dyslipidemia and diabetes leads to a stepwise increased risk for short-term mortality in hospitalized COVID-19 patients independent of age and sex. Further studies investigating how these risk factors disproportionately affect COVID-19 patients are warranted.

Strengths and limitations of this study

- While previous data reported a high prevalence of CVD risk factors in COVID-19 patients, this study investigated whether diabetes, dyslipidemia and hypertension predict adverse outcomes.
- This study is limited by the use of medication as surrogate for cardiovascular risk factors
- The causality of the investigated risk factors remains to be addressed in future studies.

For peer review only - http://bmjopen.bmj.com/site/about/guidelines.xhtml

Introduction

The global spread of coronavirus disease 2019 (COVID-19), first identified in Wuhan, China, in December 2019, has ignited an unprecedented ongoing global pandemic.(1) Although most infected individuals experience only mild symptoms that do not require hospitalization, the absolute number of patients requiring hospital admission is staggering. Risk stratification of these patients is crucial to optimize the use of hospital resources.(2) Several associations with adverse outcomes in COVID-19 patients have been identified, including factors that also predispose to cardiovascular disease (CVD), such as older age, male sex, hypertension, overweight and diabetes.(3,4) Furthermore, individuals with overt CVD appear to be affected more seriously by COVID-19 infection.(5)

The association between cardiovascular events and infectious diseases is well established. Examples include the increased prevalence of myocardial infarction during influenza pandemics,(6) and the higher number of cardiac complications in patients hospitalized for community-acquired pneumonia.(7) However, there are conflicting data whether the presence of shared CVD and COVID-19 risk factors merely reflect advanced age and history of ischemic heart disease in patients who develop severe infection, or are independently associated with adverse outcomes in the COVID-19 patient population.(4,8) For example, a higher than expected prevalence of diabetes, hypertension, obesity, and history of CVD was reported during the previous outbreak of the Middle East respiratory syndrome coronavirus (MERS-CoV), which shares many similarities with COVID-19.(9)

In the present study, we hypothesized that three major risk independent CVD risk factors are associated with adverse outcomes in COVID-19 patients. To this end, we evaluated the time-to-event relationship between COVID-19 disease outcomes and a history of medication use for hypertension, dyslipidemia, and diabetes mellitus in a large prospective Dutch cohort of hospitalized COVID-19 patients.

For peer review only

For peer review only - http://bmjopen.bmj.com/site/about/guidelines.xhtml

Patients and Methods

Study design

CovidPredict is a Dutch multicenter initiative to collect data of hospitalized patients with confirmed COVID-19.(10) For this study, patients from 8 participating hospitals, including two university hospitals were included. All hospitalized patients >18 years with a positive COVID-19 polymerase chain reaction (PCR) or high suspicion based on CT-imaging of the thorax were included (CO-RADS score 4 or 5).(11,12) A waiver for the use hospital record data was obtained from the Medical Ethical Committees of the participating centers. Patients were given the opportunity to opt out.

The collected data was updated with daily reports on vital signs, laboratory results, complications, and clinical outcomes. In addition, the use of antihypertensive, lipid-lowering, and/or antidiabetic medication was determined from the home medication list. These were used as surrogates for hypertension, dyslipidemia, and diabetes. Antihypertensive medication was categorized as using either 0, 1, more than 1 of the following categories: non-dihydropyridine calcium channel blockers (CCBs), renin angiotensin system (RAS)-inhibitors (either angiotensin receptor antagonist or angiotensin receptor blockers) and diuretics (either loop diuretics, thiazide or thiazide like diuretics or potassium channel blockers). Lipid-lowering therapy was classified as the use of statin, ezetimibe, fibrates, or PCSK9-inhibitors. Antidiabetic medication was classified as using either 0, 1 or more than 1 of the following classes: metformin, sulfonylurea derivates, GLP-1 agonists, DDP4- inhibitors, SGLT-2 inhibitors, and insulin. Obesity was defined as a BMI >30 kg/m. Smoking was categorized into current or non/former smoker. The combined use of beta-blockers and platelet aggregation inhibitors was used as a surrogate for history of ischemic cardiac disease.

For peer review only - http://bmjopen.bmj.com/site/about/guidelines.xhtml

Outcomes were determined 3 and 6 weeks after admission, or earlier when the patient died or was discharged from the hospital.

Primary and secondary outcomes

The primary outcome consisted of overall mortality during the first 21 days following admission. Overall mortality was defined as either mortality during admission or discharge for palliative care, either at home or a palliative care facility. If the patient was discharged alive from the hospital and no further follow-up data was available, we considered the patient to be event free for the whole study period. Secondary outcomes consisted of ICU-admission and mortality in the subset of patients who had been admitted to the ICU.

Statistical analysis

For the analysis, we included all consecutive patients who were primarily admitted to one of the participating centers between February 27th and July 4th 2020. Patients with unknown medication use prior to hospitalization were excluded. Patients were categorized based on the presence of either 0, 1 or more than 1 medication based cardiovascular risk factor (hypertension, dyslipidemia, diabetes). Baseline characteristics were depicted as mean ± standard deviation for normally distributed data, nonnormally as median [interquartile range], or as number (percentage) for categorical variables, and were compared using the appropriate tests (ANOVA, Kruskal-Wallis, chi-squared). The relationship between outcomes and cumulative cardiovascular risk factors was determined using a Kaplan-Meier analysis. We then determined hazard ratios using Cox-regression after adjustment for age and sex, and with additional adjustment for obesity, smoking and history of ischemic cardiac disease. For ICU-mortality, a landmark analysis was performed starting from ICU-admission. Next, with Cox-regression models using the same covariates we determined the association between mortality

For peer review only - http://bmjopen.bmj.com/site/about/guidelines.xhtml

and the use of antihypertensive, lipid-lowering, and antidiabetic drugs. The proportional hazard assumptions were verified using Schoenfeld residuals. All statistical analyses were conducted with R version 3.6.3 (R Foundation, Vienna, Austria) using the Survival version 3.1-11 and Tableone version 0.11.1 packages.

Patient and public involvement

This study was performed during the first wave of the COVID-19 pandemic in the Netherlands. Therefore, it was not possible to involve patients or the public in the design, or conduct, or reporting, or dissemination plans of our research. All CovidPredict study results will be communicated via www.covidpredict.org.

For peer review only

Results

Patient characteristics

Between February 27th 2020 and July 4th 2020, a total of 1614 patients with a confirmed COVID-19 infection were primarily admitted to one of the participating centers in the Netherlands. After exclusion of patients with an unknown medication list, we included 1604 patients in the present analysis (Table 1). Their mean age was 66±15 years, 67.6% were Caucasian, and 60.5% were men. 6.5% of the admitted patients were current smokers. 1526 patients (95.7%) had a positive PCR for COVID-19 during hospitalization. The majority of admitted patients (924, 57.6%) used some form of cardiovascular medication prior to admission. Antihypertensive medication was used by 721 (45.0%) patients, of whom 497 (68.9%) used RAS-inhibitors, 256 (35.5%) calcium-antagonists, and 374 (51.9%) diuretics. Lipid-lowering therapy was used by 557 (34.7%) patients, predominantly consisting of statins (540, 96.9%). In total, 354 (22.1%) patients used antidiabetic medication, of whom 282 (79.7%) used metformin and 137 patients (38.7%) insulin. 167 patients (10.4%) used the

For peer review only - http://bmjopen.bmj.com/site/about/guidelines.xhtml

combination of a beta-blocker and a platelet aggregation inhibitor, reflecting a history of ischemic cardiac disease. In the subset of 566 patients for whom a more detailed medical history was available, we found a similar prevalence of cardiovascular disease, with 13.1% of the patients having a history of coronary artery disease, 3.7% of heart failure, 7.4% of stroke and 1.6% of peripheral arterial disease (see Supplement 1).

For peer review only

For peer review only - http://bmjopen.bmj.com/site/about/guidelines.xhtml

Cardiovascular risk factors as markers for mortality and ICU-admission

In the entire cohort, 308 (19.2%) of the patients died or were discharged for palliative care. In total 273 (17.0%) of the patients were admitted to the ICU, of whom 78 died. 1100 (68.6%) patients were discharged alive from the hospital, 50 (3.1%) were transferred to another hospital. The remaining patients were still admitted to the hospital at time of the data collection (126; 7.9%) or follow-up data was not available yet (20; 1.25%). The Kaplan-Meier analysis showed a significant association between cardiovascular risk factors and overall mortality ($p<0.0001$; Figure 1A), with a 21-day mortality of respectively 11.9%, 20.5% and 29.1% for patients with 0, 1, and ≥2 CVD-risk factors. We found no association between cardiovascular risk factors and ICU-admission ($p=0.85$; Figure 1B), with an unadjusted admission rate of respectively 17.3%, 16.6% and 18.2%. In patients admitted to the ICU, we found a trend towards increased mortality, with an ICU-mortality of 27.2%, 29.1%, 40.1% for patients with 0, 1, and ≥2 CVD-risk factors ($p=0.055$; Figure 1C). In Cox-regression analysis, a 5-year age increase was associated with a HR of 1.37 (CI 1.31-1.45) for mortality, while there was no significant association with sex (HR 1.02, CI 0.81-1.28). The presence of two or more cardiovascular risk factors was significantly associated with overall mortality (HR 1.52, 95%CI 1.15-2.02), but not with ICU-admission or ICU-mortality (Table 2). After additional adjustment for smoking, obesity, and the combined use of beta-blockers and platelet aggregation inhibitors the presence of two or more risk factors remained associated with mortality (HR 1.38, 95%CI 1.02-1.86),), while there was no increased risk in the group with 1 CVD-risk factor (HR 1.01, 95%CI 0.73-1.39).

Individual risk factors

In the Cox-regression models adjusted for age and sex, we observed that the use of two or more different classes of antihypertensives and antidiabetics were associated with 21-day

For peer review only - http://bmjopen.bmj.com/site/about/guidelines.xhtml

mortality, with HR of respectively of 1.46 (95%CI 1.11-1.91) and 2.09 (95%CI 1.55-2.80). Similarly, we found a HR of 1.25 (95%CI 0.99-1.56) for the use of lipid-lowering medication. Additional adjustment for smoking, obesity and the combined use of beta-blockers and antiplatelet medication attenuated the association between the use of BP-lowering and lipid-lowering medication towards 1.33 (95%CI 1.01-1.76) and 1.14 (95%CI 0.89-1.45) for the use of ≥2 BP-lowering drugs or ≥1 lipid-lowering drug respectively. The association between the use of 2 or more glucose-lowering medications and mortality remained significant with an adjusted HR of 1.93 (95%CI 1.43-2.62; Table 3). The hazard ratios for the other covariates included in the regression analysis can be found in Supplement 2.

Discussion

In a large Dutch cohort of hospitalized COVID-19 patients, we observed that patients with more than one risk factor for CVD had a 52% higher 3-week mortality risk, independent of age and sex. In addition, our data show that the use of two or more antihypertensives or antidiabetics, or one lipid-lowering drug is associated with adverse outcomes in COVID-19 patients. Patients using two or more antidiabetic drugs had the highest mortality risk. This suggests that patients with a history of or at high risk for cardiovascular disease have an increased risk for adverse COVID-19 disease outcomes.

The prevalence of medication use for CVD risk factors was higher in COVID-19 patients than in previously described cohorts representative for the general Dutch population of similar age.(13) Wuhan-based COVID-19 cohorts were the first to describe a higher mortality in those with hypertension and diabetes.(14) However, the average age and reported prevalence of CVD risk factors was much lower in these cohorts than in the present study.(4) The increased case-fatality rate in Europe and the US compared with China may in part be

For peer review only - http://bmjopen.bmj.com/site/about/guidelines.xhtml

attributable to demographic differences in the COVID-19 infected population.(15) Recent European and US-based cohorts indeed demonstrate a higher average age with a similar distribution of hypertension and diabetes compared to our study, and also show a higher mortality in COVID-19 patients with hypertension and diabetes.(16,17) In line with the results from a large US-based cohort, we observed that age was significantly associated with mortality, while we found no significant difference in mortality between sexes.(18) This suggests that although men are more often hospitalized, there is no substantial difference in mortality after admission for severe COVID-19 infection. We add to these findings that the accumulation of CVD risk factors is associated with mortality, independent of age, sex, presence of coronary artery disease, smoking and obesity in hospitalized patients.

Analogous to COVID-19, CVD risk factors are also prevalent among patients hospitalized for community-acquired pneumonia.(19) However, we observed a stronger association between hypertension, diabetes, dyslipidemia and mortality in COVID-19 patients compared with previous studies on community-acquired pneumonia, despite a similar prevalence of CVD risk factors.(20) In addition, these effects remained significant after adjustment for covariates such as smoking, obesity, and the use of beta-blockers and antiplatelet drugs as surrogate for a history of ischemic cardiac disease. This might suggest that CVD risk factors in COVID-19 patients disproportionally affect the clinical course of COVID-19 patients compared to other infectious diseases. The present findings are comparable to the previous MERS-CoV outbreak, which also saw a preponderance of hypertension and diabetes in hospitalized patients.(9) Because both coronaviruses enter the cell through the angiotensin converting enzyme 2 (ACE2) receptor, a hypothesis has become that upregulation of ACE2 in patients with hypertension and/or diabetes facilitates transmission of the virus.(21–24) However, recent studies have shown no association between the use of RAS-medication and the disease

For peer review only - http://bmjopen.bmj.com/site/about/guidelines.xhtml

For peer review only

course of COVID-19.(25,26) Furthermore, this theory does not explain the increased risk of mortality paired with other CVD risk factors in COVID-19 patients. An alternative explanation is that CVD risk factors predispose to myocardial injury in COVID-19 infected patients, which is likely to contribute to a more severe clinical course.(27,28) Two recent studies revealed viral RNA in the myocardium of COVID-19 patients, suggesting that SARS-CoV-2 might infect the heart directly. It can be speculated that those with (pre-clinical) atherosclerosis are prone to experience coronary ischemia from viral myocardial involvement.(29,30)

In our cohort of COVID-19 patients, the presence of diabetes had the strongest association with mortality and remained significant after adjustment for covariates such as smoking, obesity, and the use of beta-blockers and antiplatelet drugs as surrogate for coronary artery disease. We observed smaller effect sizes for the presence of hypertension and dyslipidemia. These findings might suggest that diabetes predisposes for adverse outcomes in COVID-19 patients not only through its association with CVD, but potentially via other pathophysiological pathways specific to diabetes. A recent Chinese cohort showed diabetes to be associated with higher ICU-admission and more in-hospital mortality, but not independently of hypertension and history of CVD.(8) Interestingly, in those with diabetes, hypertension was associated with in-hospital death, independently of history of CVD, further supporting the additive effect of CVD risk factors on COVID-19 mortality. Nevertheless, it is hard to disentangle the precise relation based on epidemiological data. In line with current guidelines on CVD risk management, cardiovascular medication from different classes were often prescribed together in our cohort, also in patients with diabetes.(31) This makes it difficult to assess their separate contribution to mortality. It remains, therefore, unknown whether diabetes alone is associated with a higher risk of adverse outcomes or whether it is

For peer review only - http://bmjopen.bmj.com/site/about/guidelines.xhtml

merely a reflection of increased vascular ageing in combination with the other risk factors.(32) In contrast to our results, the recent cohort of Cummings et al, found that among cardiovascular risk factors, only chronic cardiac disease was a strong predictor for hospital mortality, while smaller associations were found for other risk factors, including diabetes.(18) In line, a recent Italian cohort of hospitalized COVID-19 patients showed an increased prevalence of hypertension and diabetes amongst non-survivors, but only diabetes was an independent predictor after adjustment for other comorbidities.(33) In the present study, the cumulative presence of CVD risk factors did not show an association with increased risk for ICU-admission. This may have been influenced by selection prior to ICU admission, where the presence of co-morbidity was taken into account in the shared decision-making process, leading to a relative underrepresentation of patients with CVD risk factors.

The present analysis has several limitations. First, data collection was based on data collection forms of the WHO, which did not include detailed information on cardiovascular disease history. For this reason, we relied on medication use as a surrogate marker for established cardiovascular risk factors or disease, which has been used before in big cohort studies.(34) Nevertheless, some of these drugs might have been prescribed for different indications, The current study was not designed to assess the relationship between specific drugs and COVID-19 outcomes. Secondly, we only obtained follow-up during the first 21 days, however as depicted in the Kaplan-Meier analysis, almost all events occurred during the first 14 days, in line with earlier descriptions.(4) Finally, we cannot exclude that mortality in the current study is partially caused by other factors than COVID-19. However, as we used 21-day mortality as our primary outcome and only included patients admitted to the hospital with confirmed COVID-19 infection, it is very likely that the majority of deaths were directly attributable to COVID-19.

For peer review only

In conclusion, the accumulation of CVD risk factors leads to a stepwise increased risk for short-term mortality in hospitalized COVID-19 patients. Patients with diabetes had the highest risk, followed by similar risks for hypertension and dyslipidemia. Mechanistic studies investigating how CVD risk factors disproportionately affect COVID-19 patients compared to other infectious diseases are warranted.

Acknowledgements

We would like to thank the CovidPredict consortium (www.covidpredict.org) for their efforts in providing the patient data.

Author contributions

DC, NN, YK, LR, ES, BJvdB, LV, MB, PE conceptualized and designed the study. TD, HM, SS, RD, AE, AR contributed substantially to the acquisition of data. DC, NN, YK, LR performed the data analysis. DC, NN, YK, LR, ES, BJvdB drafted the manuscript. TD, HM, SS, RD, AE, AR, PE, MB, LV critically revised the manuscript. All authors have provided final approval of the version to be submitted and agree to be accountable for all aspects of the work in ensuring that questions related to the accuracy or integrity of any part of the work are appropriately investigated and resolved..

Funding

DC is supported by a ZonMW grant (project number: 10430022010002).

Competing interests

For peer review only - http://bmjopen.bmj.com/site/about/guidelines.xhtml

N.S.N. and L.F.R. are co-founders of Lipid Tools. E.S.G.S. reports personal fees from Amgen, personal fees from Sanofi-Regeneron, personal fees from Esperion, grants from Athera, outside the submitted work.

Data availability statement

The data that support the findings of this study are available from the corresponding author, upon reasonable request.

For peer review only

For peer review only - http://bmjopen.bmj.com/site/about/guidelines.xhtml

References

1. World Health Organization. Coronavirus disease 2019 (COVID-19), situation report 92.
2. Lipsitch M, Swerdlow DL, Finelli L. Defining the Epidemiology of Covid-19 - Studies Needed. N Engl J Med. 2020 Mar;382(13):1194–6.
3. Wilkins JT, Ning H, Berry J, Zhao L, Dyer AR, Lloyd-Jones DM. Lifetime risk and years lived free of total cardiovascular disease. JAMA. 2012;308(17):1795–801.
4. Zhou F, Yu T, Du R, Fan G, Liu Y, Liu Z, et al. Clinical course and risk factors for mortality of adult inpatients with COVID-19 in Wuhan, China: a retrospective cohort study. Lancet. 2020;
5. Zheng YY, Ma YT, Zhang JY, Xie X. COVID-19 and the cardiovascaular system. Nat Rev Cardiol. 2020 Mar;17:259–60.
6. Nguyen JL, Yang W, Ito K, Matte TD, Shaman J, Kinney PL. Seasonal influenza infections and cardiovascular disease mortality. JAMA Cardiol. 2016 Jun;1(3):274–81.
7. Corrales-Medina VF, Musher DM, Wells GA, Chirinos JA, Chen L, Fine MJ. Cardiac complications in patients with community-acquired pneumonia incidence, timing, risk factors, and association with short-term mortality. Circulation. 2012 Feb;125(6):773–81.
8. Shi Q, Zhang X, Jiang F, Zhang X, Hu N, Bimu C, et al. Clinical Characteristics and Risk Factors for Mortality of COVID-19 Patients With Diabetes in Wuhan, China: A Two-Center, Retrospective Study. Diabetes Care. 2020;43(7):1382–91.
9. Badawi A, Ryoo SG. Prevalence of comorbidities in the Middle East respiratory syndrome coronavirus (MERS-CoV): a systematic review and meta-analysis. Int J Infect Dis. 2016 Aug;49:129–33.
10. Peters EJG, Collard D, Van Assen S, Beudel M, Bomers MK, Buijs J, et al. Outcomes

For peer review only - http://bmjopen.bmj.com/site/about/guidelines.xhtml

of persons with COVID-19 in hospitals with and without standard treatment with (Hydroxy)chloroquine. Clin Microbiol Infect. 2020;(xxxx):0–4.

11. Bai HX, Hsieh B, Xiong Z, Halsey K, Choi JW, Tran TML, et al. Performance of radiologists in differentiating COVID-19 from viral pneumonia on chest CT. Radiology. 2020;200823.

12. Prokop M, Van Everdingen W, Van Rees Vellinga T, Van Ufford HQ, Stöger L, Beenen L, et al. CO-RADS: A Categorical CT Assessment Scheme for Patients Suspected of Having COVID-19-Definition and Evaluation. Radiology. 2020;296(2):E97–104.

13. van Laer SD, Snijder MB, Agyemang C, Peters RJG, van den Born BJH. Ethnic differences in hypertension prevalence and contributing determinants – the HELIUS study. Eur J Prev Cardiol. 2018;25(18):1914–22.

14. Guan WJ, Ni ZY, Hu Y, Liang WH, Ou CQ, He JX, et al. Clinical Characteristics of Coronavirus Disease 2019 in China. N Engl J Med. 2020;1–13.

15. Onder G, Rezza G, Brusaferro S. Case-Fatality Rate and Characteristics of Patients Dying in Relation to COVID-19 in Italy. JAMA - J Am Med Assoc. 2020;2019:2019–20.

16. Richardson S, Hirsch JS, Narasimhan M, Crawford JM, McGinn T, Davidson KW, et al. Presenting Characteristics, Comorbidities, and Outcomes Among 5700 Patients Hospitalized With COVID-19 in the New York City Area. Jama. 2020;10022:1–8.

17. Goyal P, Choi JJ, Pinheiro LC, Schenck EJ, Chen R, Jabri A, et al. Clinical Characteristics of Covid-19 in New York City. N Engl J Med. 2020 Apr 17;

18. Cummings MJ, Baldwin MR, Abrams D, Jacobson SD, Meyer BJ, Balough EM, et al. Epidemiology, clinical course, and outcomes of critically ill adults with COVID-19 in New York City: a prospective cohort study. Lancet [Internet]. 2020;S0140-

For peer review only - http://bmjopen.bmj.com/site/about/guidelines.xhtml

6736(20)31189-2. Available from: http://dx.doi.org/10.1016/S0140-6736(20)31189-2

19. Corrales-Medina VF, Musher DM, Shachkina S, Chirinos JA. Acute pneumonia and the cardiovascular system. Lancet. 2013;381(9865):496–505.

20. Violi F, Cangemi R, Falcone M, Taliani G, Pieralli F, Vannucchi V, et al. Cardiovascular complications and short-term mortality risk in community-acquired pneumonia. Clin Infect Dis. 2017;64(11):1486–93.

21. Fang L, Karakiulakis G, Roth M. Are patients with hypertension and diabetes mellitus at increased risk for COVID-19 infection? Lancet Respir Med. 2020 Apr;8(4):e21.

22. Vaduganathan M, Vardeny O, Michel T, McMurray JJ V, Pfeffer MA, Solomon SD. Renin-Angiotensin-Aldosterone System Inhibitors in Patients with Covid-19. N Engl J Med. 2020 Mar;

23. Kuster GM, Pfister O, Burkard T, Zhou Q, Twerenbold R, Haaf P, et al. SARS-CoV2: should inhibitors of the renin–angiotensin system be withdrawn in patients with COVID-19? Eur Heart J. 2020 Mar;

24. Danser AHHJ, Epstein M, Batlle D. Renin-Angiotensin System Blockers and the COVID-19 Pandemic: At Present There Is No Evidence to Abandon Renin-Angiotensin System Blockers. Hypertension. 2020 Mar;

25. Mackey K, King VJ, Gurley S, Kiefer M, Liederbauer E, Vela K, et al. Risks and Impact of Angiotensin-Converting Enzyme Inhibitors or Angiotensin-Receptor Blockers on SARS-CoV-2 Infection in Adults. Ann Intern Med [Internet]. 2020 May 15; Available from: https://doi.org/10.7326/M20-1515

26. Mancia G, Rea F, Ludergnani M, Apolone G, Corrao G. Renin–angiotensin–aldosterone system blockers and the risk of COVID-19. N Engl J Med. 2020;382(25):2431–40.

27. Shi S, Qin M, Shen B, Cai Y, Liu T, Yang F, et al. Association of Cardiac Injury with

For peer review only - http://bmjopen.bmj.com/site/about/guidelines.xhtml

Mortality in Hospitalized Patients with COVID-19 in Wuhan, China. JAMA Cardiol. 2020;

28. Atallah B, Mallah SI, AbdelWareth L, AlMahmeed W, Fonarow GC. A marker of systemic inflammation or direct cardiac injury: should cardiac troponin levels be monitored in COVID-19 patients? Eur Hear journal Qual care Clin outcomes. 2020;6(3):204–7.

29. Wichmann D, Sperhake J-P, Lütgehetmann M, Steurer S, Edler C, Heinemann A, et al. Autopsy Findings and Venous Thromboembolism in Patients With COVID-19. Ann Intern Med [Internet]. 2020 May 6; Available from: https://doi.org/10.7326/M20-2003

30. Schaller T, Hirschbühl K, Burkhardt K, Braun G, Trepel M, Märkl B, et al. Postmortem Examination of Patients with COVID-19. JAMA - J Am Med Assoc. 2020;323(24):2518–20.

31. The Task Force for the management of arterial hypertension of the European Society of Cardiology (ESC) and the European Society of Hypertension (ESH). 2018 ESC/ESH Guidelines for the management of arterial hypertension. Eur Heart J. 2018 Aug 25;39(33):3021–104.

32. Ungvari Z, Tarantini S, Donato AJ, Galvan V, Csiszar A. Mechanisms of vascular aging. Circ Res. 2018;123(7):849–67.

33. Iaccarino G, Grassi G, Borghi C, Ferri C, Salvetti M, Volpe M. Age and Multimorbidity Predict Death Among COVID-19 Patients: Results of the SARS-RAS Study of the Italian Society of Hypertension. Hypertens (Dallas, Tex 1979). 2020;76(2):366–72.

34. Attaei MW, Khatib R, McKee M, Lear S, Dagenais G, Igumbor EU, et al. Availability and affordability of blood pressure-lowering medicines and the effect on blood pressure control in high-income, middle-income, and low-income countries: an analysis of the

For peer review only

For peer review only - http://bmjopen.bmj.com/site/about/guidelines.xhtml

PURE study data. Lancet Public Heal. 2017;2(9):e411–9.

For peer review only

For peer review only - http://bmjopen.bmj.com/site/about/guidelines.xhtml

Figure legends

Figure 1. Survival and time-to event analysis of cumulative cardiovascular risk factors on mortality, ICU-admission and ICU-mortality

Kaplan-Meier analysis of hypertension, dyslipidemia and diabetes stratified into 0, 1 or more risk factors versus adverse clinical outcomes. Panel (A) depicts mortality, panel (B) ICU-admission. Panel (C) depicts ICU-mortality, for which a landmark analysis following ICU-admission was performed. Log-rank test was used to test for differences between curves. RF, risk factor; ICU, intensive care unit.

For peer review only

Table 1. Baseline characteristics

	Overall	0 RF	1 RF	≥2 RF	p
n	1604	680	400	524	
Age (mean (SD))	65.67 (15.06)	58.59 (15.71)	69.93 (13.01)	71.62 (11.45)	<0.001
Women	633 (39.5)	298 (43.8)	170 (42.5)	165 (31.5)	<0.001
Chronic cardiac disease	467 (29.2)	71 (10.5)	117 (29.3)	279 (53.4)	<0.001
Hypertension	734 (46.0)	80 (11.8)	242 (60.8)	412 (78.8)	<0.001
Chronic pulmonary disease	288 (18.0)	92 (13.6)	82 (20.6)	114 (21.9)	<0.001
Asthma	179 (11.2)	77 (11.4)	41 (10.3)	61 (11.7)	0.793
Chronic kidney disease	150 (9.4)	17 (2.5)	48 (12.0)	85 (16.3)	<0.001
Diabetes	411 (25.7)	19 (2.8)	66 (16.5)	326 (62.3)	<0.001
Malignant neoplasm	98 (6.2)	36 (5.3)	25 (6.3)	37 (7.1)	0.455
Chronic hematologic disorder	57 (3.6)	32 (4.7)	11 (2.8)	14 (2.7)	0.1
Smoking	78 (6.5)	30 (6.0)	16 (5.4)	32 (7.8)	0.383
Obesity	464 (30.8)	164 (26.1)	112 (29.4)	188 (37.8)	<0.001
Combined use of beta-blockers and antiplatelet drugs	167 (10.4)	7 (1.0)	35 (8.8)	125 (23.9)	<0.001
Antihypertensive-Rx					<0.001
0	883 (55.0)	680 (100.0)	145 (36.2)	58 (11.1)	
1	377 (23.5)	0 (0.0)	141 (35.2)	236 (45.0)	
2	282 (17.6)	0 (0.0)	95 (23.8)	187 (35.7)	
≥ 3	62 (3.9)	0 (0.0)	19 (4.8)	43 (8.2)	
Lipid-lowering-Rx					<0.001
0	1047 (65.3)	0 (0.0)	296 (74.0)	89 (13.5)	
≥ 1	557 (34.7)	0 (0.0)	104 (26.0)	453 (86.5)	
Glucose-lowering-Rx					<0.001
0	1250 (77.9)	680 (100.0)	359 (89.8)	211 (40.3)	
1	170 (10.6)	0 (0.0)	23 (5.8)	147 (28.1)	
≥ 2	184 (11.5)	0 (0.0)	18 (4.5)	166 (31.7)	

RF, risk factor; SD, standard deviation; IQR, interquartile range; Rx, medication. P-values indicate comparison between subgroups based on the presence of cumulative risk factors.

For peer review only - http://bmjopen.bmj.com/site/about/guidelines.xhtml

Table 2. Effect of cumulative cardiovascular risk factors on primary and secondary outcomes

	Cumulative risk factors	Model 1				Model 2			
		HR	95%CI		p	HR	95%CI		p
Mortality	0 RF	1 (ref)				1 (ref)			
	1 RF	1.04	0.76	1.43	0.786	1.01	0.73	1.39	0.956
	≥2 RF	1.52	1.15	2.02	0.004	1.38	1.02	1.86	0.034
ICU admission	0 RF	1 (ref)				1 (ref)			
	1 RF	1.11	0.80	1.53	0.534	1.06	0.77	1.47	0.723
	≥2 RF	1.15	0.85	1.56	0.355	1.07	0.77	1.48	0.681
ICU-mortality	0 RF	1 (ref)				1 (ref)			
	1 RF	0.86	0.46	1.60	0.625	1.01	0.73	1.39	0.957
	≥2 RF	1.52	0.90	2.55	0.115	1.38	1.02	1.86	0.035

Cox-regression for the effect of cumulative cardiovascular risk factors on mortality, ICU-admission and ICU-mortality, after adjustment for sex and age (model 1), and with additional adjustments for obesity, smoking and history of coronary artery disease (model 2). HR, hazard ratio; CI, confidence interval; ICU, intensive care unit.

For peer review only

Table 3. Effect of antihypertensive, lipid-lowering, and antidiabetic medications on mortality

	Model 1				Model 2			
**Mortality**	**HR**	**95%CI**		**p**	**HR**	**95%CI**		**p**
0 antihypertensive-Rx	1 (ref)				1 (ref)			
1 antihypertensive-Rx	1.08	0.81	1.43	0.597	1.04	0.78	1.38	0.79
≥2 antihypertensive-Rx	1.46	1.11	1.91	0.006	1.33	1.01	1.76	0.043
0 lipid-lowering-Rx	1 (ref)				1 (ref)			
≥1 lipid-lowering-Rx	1.25	0.99	1.56	0.058	1.14	0.89	1.45	0.292
0 antidiabetic-Rx	1 (ref)				1 (ref)			
1 antidiabetic-Rx	1.34	0.97	1.85	0.077	1.25	0.9	1.74	0.182
≥2 antidiabetic-Rx	2.09	1.55	2.80	<0.001	1.93	1.43	2.62	<0.001

Cox-regression for the effect of the number of antihypertensive, lipid-lowering and antidiabetic drug classes on 21-day mortality, defined as either hospital mortality or discharge for palliative care, with adjustment for age and sex (model 1), and with additional adjustments for obesity, smoking and history of coronary artery disease (model 2). Rx, medication; HR, hazard ratio; CI, confidence interval.

For peer review only

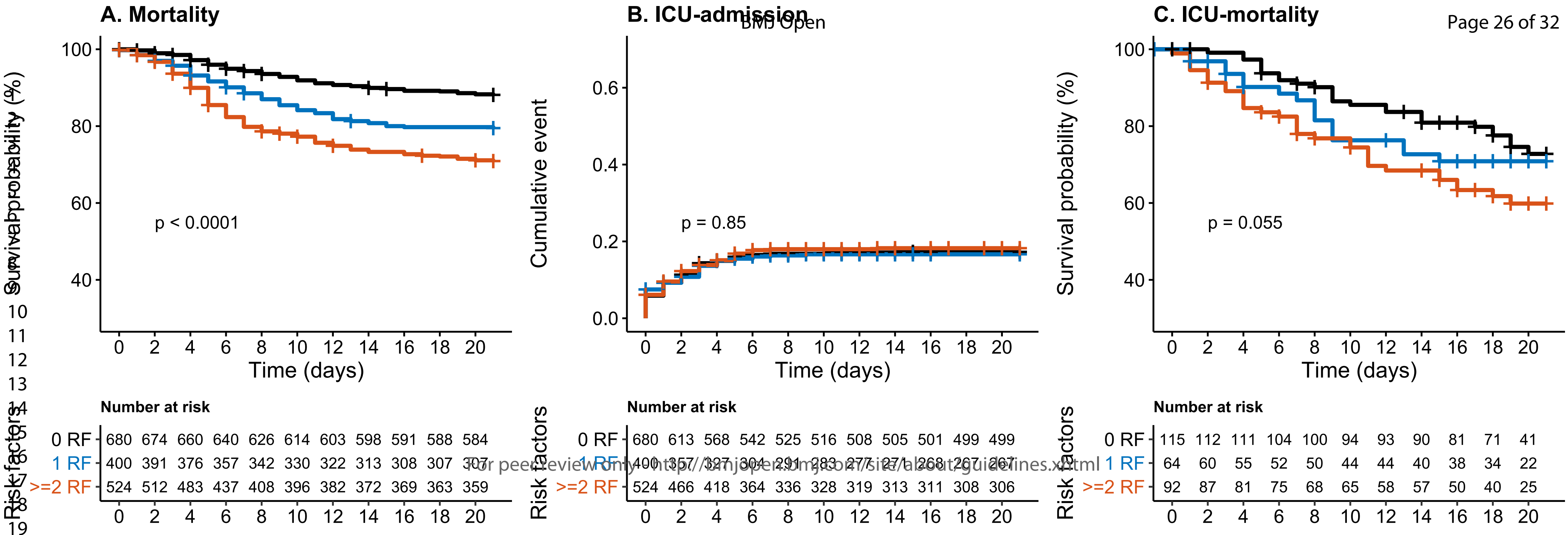

For peer review only - http://bmjopen.bmj.com/site/about/guidelines.xhtml

Supplement 1: Baseline characteristics in subset with detailed clinical history

In three of the participating hospitals (Amsterdam UMC location AMC, Amsterdam UMC location VUMC, Flevoziekenhuis), we collected additional clinical information to validate the medication use as surrogate marker for cardiovascular risk factors and disease, depicted in the table below.

For peer review only

Supplementary table: Baseline characteristics of complete cohort, and subgroup where additional information about prior cardiovascular events was available.

	Overall	Subset
n	1604	566
Age (mean (SD))	65.67 (15.06)	61.37 (14.60)
Women	633 (39.5)	241 (42.6)
Chronic cardiac disease	467 (29.2)	125 (22.1)
Hypertension	734 (46.0)	252 (44.8)
Chronic pulmonary disease	288 (18.0)	77 (13.6)
Asthma	179 (11.2)	56 (9.9)
Chronic kidney disease	150 (9.4)	57 (10.1)
Diabetes	411 (25.7)	154 (27.2)
Malignant neoplasm	98 (6.2)	31 (5.5)
Chronic hematologic disorder	57 (3.6)	23 (4.1)
Smoking	78 (6.5)	28 (6.2)
Obesity	464 (30.8)	168 (32.8)
Combined use of beta-blockers and antiplatelet drugs	167 (10.4)	50 (8.8)
History of coronary artery disease		74 (13.1)
History of heart failure		21 (3.7)
History of stroke		42 (7.4)
History of peripheral artery disease		9 (1.6)
Antihypertensive-Rx		
0	883 (55.0)	327 (57.8)
1	377 (23.5)	126 (22.3)
2	282 (17.6)	81 (14.3)
≥ 3	62 (3.9)	32 (5.7)
Lipid-lowering-Rx		
0	1047 (65.3)	399 (70.5)
≥ 1	557 (34.7)	167 (29.5)
Glucose-lowering-Rx		
0	1250 (77.9)	435 (76.9)
1	170 (10.6)	60 (10.6)
≥ 2	184 (11.5)	71 (12.5)

RF, risk factor; SD, standard deviation; IQR, interquartile range, Rx medication.

Cox-regression for the effect of the number of antihypertensive, lipid-lowering and antidiabetic drug classes on mortality. Table depicts results from multivariate analysis

For peer review only

including age, sex, obesity, current smoker and the use of beta-blockers and antiplatelet medication. Rx, medication; HR, hazard ratio; CI, confidence interval.

For peer review only

Supplement 2: Cox-regression models with additional correction for smoking, obesity and the use of both a beta-blocker and antiplatelet drug.

We performed an additional analysis for the association between the cardiovascular risk factors and mortality with adjustment for smoking, obesity and the use of both a beta-blocker and antiplatelet drug. Data for smoking status was missing for 22.7% of the patients, data for obesity was missing for 9.2% of the patients. Imputation for these covariates was performed using the Multivariate Imputation by Chained (mice) package version 3.8.0. The pooled results averaged over 25 iterations are depicted in the table below. A complete cases analysis showed similar results (data not shown).

For peer review only

Supplementary table: Effect of the cumulative risk factors, antihypertensive medication, lipid-lowering medication and antidiabetic medication on mortality after adjustment for covariates.

	Mortality					IC-admission					IC-mortality			
**Covariate**	**HR**	**95%CI**		**P-value**	**Covariate**	HR	95%CI		P-value	**Covariate**	HR	95%CI		P-value
**1 RF**	1.01	0.73	1.39	0.956	**1 RF**	1.06	0.77	1.47	0.723	**1 RF**	1.01	0.73	1.39	0.957
**≥2 RF**	1.38	1.02	1.86	0.034	**≥2 RF**	1.07	0.77	1.48	0.681	**≥2 RF**	1.38	1.02	1.86	0.035
**Women**	0.94	0.74	1.19	0.612	**Women**	0.45	0.34	0.59	<0.001	**Women**	0.94	0.74	1.19	0.612
**Age**	1.07	1.06	1.08	<0.001	**Age**	0.99	0.98	1.00	0.023	**Age**	1.07	1.06	1.08	<0.001
**Beta-blockers and antiplatelet-Rx**	1.27	0.93	1.73	0.13	**Beta-blockers and antiplatelet-Rx**	1.10	0.73	1.67	0.648	**Beta-blockers and antiplatelet-Rx**	1.27	0.93	1.73	0.131
**Obesity**	1.26	0.97	1.65	0.086	**Obesity**	1.31	1.01	1.71	0.042	**Obesity**	1.26	0.97	1.65	0.087
**Current smoker**	0.83	0.46	1.5	0.533	**Current smoker**	0.66	0.37	1.19	0.167	**Current smoker**	0.83	0.46	1.50	0.534

	Mortality					Mortality					Mortality			
**Covariate**	HR	95%CI		P-value	**Covariate**	HR	95%CI		P-value	**Covariate**	HR	95%CI		P-value
**1 BP-lowering-Rx**	1.04	0.78	1.38	0.79	**1 lipid-lowering-Rx**	1.14	0.89	1.45	0.292	**1 antidiabetic-Rx**	1.25	0.9	1.74	0.182
**≥2 BP-lowering-Rx**	1.33	1.01	1.76	0.043						**≥2 antidiabetic-Rx**	1.93	1.43	2.62	<0.001
**Women**	0.9	0.72	1.14	0.391	**Women**	0.92	0.73	1.17	0.502	**Women**	0.93	0.74	1.17	0.538
**Age**	1.07	1.06	1.08	<0.001	**Age**	1.07	1.06	1.08	<0.001	**Age**	1.07	1.06	1.08	<0.001
**Beta-blockers and antiplatelet-Rx**	1.34	0.99	1.82	0.056	**Beta-blockers and antiplatelet-Rx**	1.36	1	1.86	0.051	**Beta-blockers and antiplatelet-Rx**	1.34	0.99	1.8	0.057
**Obesity**	1.27	0.97	1.66	0.078	**Obesity**	1.3	1	1.69	0.051	**Obesity**	1.18	0.9	1.55	0.226
**Current smoker**	0.87	0.5	1.51	0.615	**Current smoker**	0.87	0.5	1.52	0.624	**Current smoker**	0.91	0.52	1.58	0.725

For peer review only - http://bmjopen.bmj.com/site/about/guidelines.xhtml

STROBE 2007 (v4) Statement—Checklist of items that should be included in reports of *cohort studies*

Section/Topic	Item #	Recommendation	Reported on page #
**Title and abstract**	1	(*a*) Indicate the study's design with a commonly used term in the title or the abstract	1
		(*b*) Provide in the abstract an informative and balanced summary of what was done and what was found	2
**Introduction**			
Background/rationale	2	Explain the scientific background and rationale for the investigation being reported	4
Objectives	3	State specific objectives, including any prespecified hypotheses	4
**Methods**			
Study design	4	Present key elements of study design early in the paper	5
Setting	5	Describe the setting, locations, and relevant dates, including periods of recruitment, exposure, follow-up, and data collection	5
Participants	6	(*a*) Give the eligibility criteria, and the sources and methods of selection of participants. Describe methods of follow-up	5
		(*b*) For matched studies, give matching criteria and number of exposed and unexposed	
Variables	7	Clearly define all outcomes, exposures, predictors, potential confounders, and effect modifiers. Give diagnostic criteria, if applicable	6
Data sources/ measurement	8*	For each variable of interest, give sources of data and details of methods of assessment (measurement). Describe comparability of assessment methods if there is more than one group	5
Bias	9	Describe any efforts to address potential sources of bias	6
Study size	10	Explain how the study size was arrived at	6
Quantitative variables	11	Explain how quantitative variables were handled in the analyses. If applicable, describe which groupings were chosen and why	6
Statistical methods	12	(*a*) Describe all statistical methods, including those used to control for confounding	6
		(*b*) Describe any methods used to examine subgroups and interactions	6
		(*c*) Explain how missing data were addressed	6; 23
		(*d*) If applicable, explain how loss to follow-up was addressed	6
		(*e*) Describe any sensitivity analyses	6
**Results**			

For peer review only

Participants	13*	(a) Report numbers of individuals at each stage of study—eg numbers potentially eligible, examined for eligibility, confirmed eligible, included in the study, completing follow-up, and analysed	7
		(b) Give reasons for non-participation at each stage	7
		(c) Consider use of a flow diagram	7
Descriptive data	14*	(a) Give characteristics of study participants (eg demographic, clinical, social) and information on exposures and potential confounders	7, 19, 27
		(b) Indicate number of participants with missing data for each variable of interest	23
		(c) Summarise follow-up time (eg, average and total amount)	6, 18, 22
Outcome data	15*	Report numbers of outcome events or summary measures over time	8
Main results	16	(*a*) Give unadjusted estimates and, if applicable, confounder-adjusted estimates and their precision (eg, 95% confidence interval). Make clear which confounders were adjusted for and why they were included	8,20, 21, 24
		(*b*) Report category boundaries when continuous variables were categorized	
		(*c*) If relevant, consider translating estimates of relative risk into absolute risk for a meaningful time period	
Other analyses	17	Report other analyses done—eg analyses of subgroups and interactions, and sensitivity analyses	8, 22
**Discussion**			
Key results	18	Summarise key results with reference to study objectives	9
**Limitations**			
Interpretation	20	Give a cautious overall interpretation of results considering objectives, limitations, multiplicity of analyses, results from similar studies, and other relevant evidence	9,10,11,12
Generalisability	21	Discuss the generalisability (external validity) of the study results	9,10,11,12
**Other information**			
Funding	22	Give the source of funding and the role of the funders for the present study and, if applicable, for the original study on which the present article is based	13

*Give information separately for cases and controls in case-control studies and, if applicable, for exposed and unexposed groups in cohort and cross-sectional studies.

Note: An Explanation and Elaboration article discusses each checklist item and gives methodological background and published examples of transparent reporting. The STROBE checklist is best used in conjunction with this article (freely available on the Web sites of PLoS Medicine at http://www.plosmedicine.org/, Annals of Internal Medicine at http://www.annals.org/, and Epidemiology at http://www.epidem.com/). Information on the STROBE Initiative is available at www.strobe-statement.org.

For peer review only
